# Responses of soil microeukaryotic communities to short-term fumigation-incubation revealed by MiSeq amplicon sequencing

**DOI:** 10.3389/fmicb.2015.01149

**Published:** 2015-10-20

**Authors:** Lin Chen, Jianming Xu, Youzhi Feng, Juntao Wang, Yongjie Yu, Philip C. Brookes

**Affiliations:** ^1^Institute of Soil and Water Resources and Environmental Science, Zhejiang UniversityHangzhou, China; ^2^State Key Laboratory of Soil and Sustainable Agriculture, Institute of Soil Science, Chinese Academy of SciencesNanjing, China; ^3^State Key Laboratory of Urban and Regional Ecology, Research Center for Eco-Environmental Sciences, Chinese Academy of SciencesBeijing, China; ^4^College of Applied Meteorology, Nanjing University of Information Science and TechnologyNanjing, China

**Keywords:** fumigation, fungi, protist, enzymes, network analysis

## Abstract

In soil microbiology, there is a “paradox” of soil organic carbon (SOC) mineralization, which is that even though chloroform fumigation destroys majority of the soil microbial biomass, SOC mineralization continues at the same rate as in the non-fumigated soil during the incubation period. Soil microeukaryotes as important SOC decomposers, however, their community-level responses to chloroform fumigation are not well understood. Using the 18S rRNA gene amplicon sequencing, we analyzed the composition, diversity, and C-metabolic functions of a grassland soil and an arable soil microeukaryotic community in response to fumigation followed by a 30-day incubation. The grassland and arable soil microeukaryotic communities were dominated by the fungal Ascomycota (80.5–93.1% of the fungal sequences), followed by the protistan Cercozoa and Apicomplexa. In the arable soil fungal community, the predominance of the class Sordariomycetes was replaced by the class Eurotiomycetes after fumigation at days 7 and 30 of the incubation. Fumigation changed the microeukaryotic α-diversity in the grassland soil at days 0 and 7, and β-diversity in the arable soil at days 7 and 30. Network analysis indicated that after fumigation fungi were important groups closely related to other taxa. Most phylotypes (especially Sordariomycetes, Dothideomycetes, Coccidia, and uncultured Chytridiomycota) were inhibited, and only a few were positively stimulated by fumigation. Despite the inhibited Sordariomycetes, the fumigated communities mainly consisted of Eurotiomycetes and Sordariomycetes (21.9 and 36.5% relative frequency, respectively), which are able to produce hydrolytic enzymes associated with SOC mineralization. Our study suggests that fumigation not only decreases biomass size, but modulates the composition and diversity of the soil microeukaryotic communities, which are capable of driving SOC mineralization by release of hydrolytic enzymes during short-term fumigation-incubation.

## Introduction

Soil microorganisms are the principal participants in most soil processes. The determination of microbial biomass can facilitate our understanding of microbial ecological functions and the magnitude of certain processes, such as soil carbon (C) and nitrogen (N) mineralization (Fierer et al., 2009). Chloroform fumigation (fumigation) is a classic method used for determination of the soil microbial biomass. Jenkinson and Powlson (1976) described a fumigation-incubation method to estimate the soil microbial biomass. They proposed that, following fumigation, the extra CO_2_ evolved from the fumigated soil compared to the similarly incubated but non-fumigated control soil during the first 10 days of incubation (termed Fumigation-incubation, FI) provides an estimate of the original soil microbial biomass (Jenkinson and Powlson, 1976). Subsequently, more analytically convenient, the fumigation-extraction method to measure microbial biomass was developed from FI (e.g., Brookes et al., 1982, 1985; Vance et al., 1987; Wu et al., 1990).

Previous investigations have observed an intriguing phenomenon that although fumigation destroyed 80–90% of the initial soil microbial biomass, following the fumigant removal, soil organic C (SOC) mineralization continued at the same rate as in the non-fumigated soil under appropriate incubation conditions for several weeks or even months (Jenkinson and Powlson, 1976; Wu et al., 1996; Kemmitt et al., 2008). Kemmitt et al. (2008) attempted to explain this phenomenon and developed the “Regulatory Gate” hypothesis. Firstly, the recalcitrant SOC was considered to be transformed into bio-available components via an abiotic process(es) (termed the “Regulatory Gate”), and this small trickle of bio-available C could then be mineralized by the soil microorganisms, independently of biomass size. Possible mechanisms of SOC transformation was considered to include chemical oxidation, chemical hydrolysis, desorption of absorbed organic matter or diffusion from within aggregates (Kemmitt et al., 2008). There could be a combination of these parameters, or, indeed, none of them (Brookes et al., 2009). There is some support for the “Regulatory Gate” hypothesis. For instance, in mineral soils, physical access to occluded or adsorbed substrates by the microbial population is the rate-limiting process governing SOC mineralization (Schimel and Schaeffer, 2012). However, when considering the “Regulatory Gate” hypothesis, we must also consider different microbial communities associated with the functioning of SOC mineralization (Paterson, 2009). The bacterial community in an arable soil subjected to fumigation, followed by inoculation with a little fresh soil, was investigated by Dominguez-Mendoza et al. (2014), who considered that some bacterial groups (e.g., Micromonosporaceae, Bacillaceae, and Paenibacillaceae) had the capacity to metabolize the fumigant-killed soil microorganisms and partially recolonize a fumigated arable soil during a 10-day incubation.

Microeukaryotes (e.g., fungi, protists, and metazoans) make important contributions to soil biogeochemical cycling and the maintenance of soil fertility because of their involvement in some key processes, such as C turnover and energy flow (Chen et al., 2012, 2014; Damon et al., 2012; Jing et al., 2014). By analyzing phospholipid fatty acids (PLFAs), Zelles et al. (1997) and Dickens and Anderson (1999) reported that the soil microeukaryotic biomass declined by 70–80% after fumigation followed by 10 and 28-day incubations. However, so far the changes in the composition, biodiversity and C-metabolic functions of the soil microeukaryotic communities are not well understood during the fumigation-incubation period. In the present study, we aimed to comprehensively survey the soil microeukaryotic communities, and further examine their changes in composition, diversity and functions in response to short-term fumigation-incubation. The following two hypotheses were tested: (i) fumigation would alter taxonomic composition and diversity patterns of the soil microeukaryotic communities, dependent on soil and incubation time, and (ii) such changed microeukaryotic communities would be still active or potentially active to drive the recalcitrant SOC mineralization. To test these hypotheses, a grassland soil was sampled from the Inner Mongolian prairie and an arable soil from Zhejiang in China. Both were fumigated with ethanol-free chloroform for 24 h, incubated aerobically for 30 days, and sampled at days 0, 7, and 30 of the incubation to determine the soil microeukaryotic community composition and diversity using a high-throughput sequencing approach. Microbial biomass, respiration rate, the metabolic quotient, potential, and specific activities of two C-acquiring enzymes (β-glucosidase and invertase) were also measured and related to the fumigated microeukaryotic communities.

## Materials and methods

### Soil description

The grassland soil was acquired from Inner Mongolia Grassland Ecosystem Research Station of Chinese Academy of Sciences located in Xilingol Region (43°33′N, 116°37′E), Inner Mongolia, China. The *Leymus chinensis* (Trin.) Tzvelev grassland has been fenced since 1980, and experiences a temperate semiarid climate, with an annual mean temperature of 0.5°C and annual average precipitation of 350 mm. The arable soil was taken from Dongyang Maize Research Institute of Zhejiang Academy of Agricultural Sciences in Dongyang County (29°27′N, 120°23′E), Zhejiang Province, China. Maize (*Zea mays* L.) has been continuously cropped twice a year for 10 years. Annual mean temperature and precipitation are 17°C and 1350 mm, respectively. The two soils were collected on September 2014, after visible plant residues and stones were removed, air-dried and sieved < 2 mm. Basal soil physiochemical index were analyzed (Table 1).

**Table 1:** **Initial soil physiochemical index**.

**Soil source**	**Soil classification (USDA)**	**Organic C^a^ (g kg^−1^)**	**Total N^b^ (g kg^−1^)**	**pH^c^ (H_2_O)**	**Clay^d^ (%)**	**Cation exchange capacity^e^ (cmol kg^−1^)**
Inner Mongolian prairie	Calcic-orthic Aridisol	26.6	3.25	7.1	21	20.88
Dongyang maize arable	Udic Cambisols	15.8	1.79	4.4	28	13.56

a Dichromate method (Nelson and Sommers, 1982).

b Kjeldahl digestion (Bremner, 1965).

c 1:2.5 soil and water suspension.

d Laser particle characterization.

e EDTA-ammonium acetate method (Lu, 2000). Cation exchange capacity is an important indicator for soil water and fertilizer-holding capacity, and soil buffering potential.

### Soil fumigation, incubation, and sampling

Soils were pre-incubated at 60% of the maximum water-holding capacity (WHC) and 25°C for 15 days, to allow microbial activity to stabilize after rewetting. Moist soil (200 g) was placed in a desiccator containing 20 ml of distilled water at the bottom (to maintain humidity), a beaker with 50 ml of ethanol-free chloroform and 50 ml of 1.0 M NaOH (to absorb CO_2_). The desiccator was evacuated until the chloroform had boiled for 3 min, and then incubated in darkness for 24 h at 25°C. The residual chloroform in the soil was then removed by repeated evacuations. The non-fumigated controls were treated similarly except that distilled water replaced ethanol-free chloroform in the desiccators and the soils were not evacuated.

Fumigated and non-fumigated soil (200 g) was transferred to stoppered 1 l glass jars, and incubated at 60% WHC and 25°C for 30 days. During the incubation period, soil moisture was controlled by weighing the jars and adding sterilized distilled water, and the air in the jars was refreshed every 2–3 days to maintain aerobic condition. Soil samples were collected at days 0 (after 24 h fumigation), 7 and 30 of the incubation. Samples were divided into two portions, one portion was stored at 4°C to determine microbial biomass C, respiration rate, invertase and β-glucosidase activities, and the other at −80°C for DNA isolation and molecular analysis.

The experiment consisted of four treatments (the grassland and arable soils with and without fumigation). All treatments were replicated three times. The grassland soil was designated “G,” the arable soil “A,” fumigation “F,” and incubation days “0, 7, and 30.”

### Determination of microbial properties

Microbial biomass C was extracted using the chloroform fumigation method (Vance et al., 1987). The C concentration was determined using a Multi C/N 3100 TOC analyzer (Analytik Jena AG, Jena, Germany), and a value of *k*_*EC*_ = 0.45 (Wu et al., 1990) was used to calibrate biomass C content. Microbial respiration rate was analyzed using the alkali absorption method, and the trapped CO_2_ concentration was measured by titration using an EasyPlus autotitrator (Mettler Toledo, Zurich, Switzerland). The metabolic quotient (*q*CO_2_) was estimated by analyzing the hourly mean CO_2_ emission per unit biomass C (Blagodatskaya and Anderson, 1998). Invertase activity was determined by a 3,5-dinitrosalicylic acid method as described by Bandick and Dick (1999).

Assay of β-glucosidase activity was adapted from Tabatabai (1994). In brief, 5.0 g of moist soil was suspended in 20 ml of modified universal buffer (pH 6.0), and 5 ml of 25 mM *p*-nitrophenyl-β-D-glucopyranoside (Aladdin, Shanghai, China) was added as the reactive substrate. The suspension was reciprocally shaken at 200 rev min^−1^ and 37°C for 1 h, and then 5 ml of 0.5 M CaCl_2_ and 20 ml of 0.1 M *Tris* buffer (pH 12.0) were added to stop substrate degradation. The solution was centrifuged at 13,000 × *g* for 1 min and the concentration of paranitrophenol (PNP) in the supernatant was measured at 400 nm on a spectrophotometer (Puyuan, Shanghai, China). The same procedure was applied to the control, except that the substrate was added after the incubation and addition of the CaCl_2_ and *Tris* buffer.

### DNA isolation, amplification, and sequencing

The total soil DNA was isolated and purified using a FastDNA spin kit (MP Biomedicals, Santa Ana, CA, USA), followed by an UltraClean DNA purification kit (MoBio, Carlsbad, CA, USA). The isolated DNA was dissolved in 50 μl of TE buffer, and the DNA quality and quantity were verified using electrophoresis on 1% agarose gels.

To produce the eukaryotic amplicon library for high-throughput sequencing, the eukaryotic 18S rRNA gene fragments were amplified using the universal primers Euk1F (5′-CTGGTTGATCCTGCCAG-3′) and Euk516R (5′-ACCAGACTTGCCCTCC-3′) (Shen et al., 2014; Shi et al., 2015). The forward and reverse primers were tagged with adapter, pad and linker sequences. Each barcode sequence (5 mer) was added to the reverse primer for pooling of multiple samples in one run of MiSeq sequencing. For each sample, PCR amplification was performed in triplicate 50-μl reaction mixtures containing 0.5 μl (125 pmol) of each forward/reverse primer, 1 μl (approximately 50 ng) of DNA template, 23 μl of ddH_2_O, and 25 μl of Premix *Taq* (Takara, Dalian, China), which consisted of 1.25 U DNA polymerase, 2 × dNTP mixture (0.4 mM), 2 × buffer (3 mM Mg^2+^), and the marker (Tartrazine/Xylene Cyanol FF). Thirty-five thermal cycles (95°C for 45 s, 56°C for 45 s, and 72°C for 1 min) were carried out with a final extension at 72°C for 7 min.

PCR amplicons pooled from the triplicate reactions were purified using a QIAquick PCR purification kit (Qiagen, Shenzhen, China), and quantified using a NanoDrop ND-1000 spectrophotometer (Thermo Scientific, Waltham, MA, USA). Equimolar amounts of amplicons from all samples (each 200 ng) were combined into a mixed sample. According to the MiSeq reagent kit preparation guide (Illumina, San Diego, CA, USA), the purified mixture was diluted and denatured to obtain the 8 pM sample DNA library and mixed with an equal volume of 8 pM PhiX (Illumina). Finally, 360 μl of the mixture library was loaded with read 1, read 2, and the index sequencing primers on a 300-cycle (2 × 150 paired ends) kit and run on a MiSeq apparatus (Illumina).

### Bioinformatics and data analysis

The 18S raw sequence data were processed using the Quantitative Insights Into Microbial Ecology (QIIME) 1.8.0-dev pipeline (Caporaso et al., 2010a) (http://qiime.org/). Poor-quality sequences (i.e., sequences of < 200 bp with an average quality score of < 25 and ambiguous characters) were discarded (Huse et al., 2007). Filtration of the sequences was done to remove erroneous operational taxonomic units (OTUs) due to sequence errors, and chimeras were detected using the UCHIME program (Edgar et al., 2011). Sequences were then binned into OTUs *de novo* at a 97% similarity level using the UCLUST algorithm (Edgar, 2010). The most highly connected sequence (i.e., the sequence with the highest similarity to all other sequences in the cluster) was chosen to represent each OTU (Hamady et al., 2008). All selected representative sequences were aligned by use of the PyNAST tool (Caporaso et al., 2010b). Taxonomy was assigned to eukaryotic phylotypes of the Silva 104 database (http://www.arb-silva.de/download/archive/qiime/). The variations in the main phylotypes induced by fumigation were expressed as log_10_-transformed odds ratio (Ganesh et al., 2014).

We obtained between 5162 and 24,947 valid sequences per sample (mean 13,471) for soil samples with the exception of a sample from G-0 treatment (Table S1). To rarify all datasets to the same level of sampling effort, 5000 sequences per sample were randomly selected for the microeukaryotic α- and β-diversity analyses. Phylogenetic diversity and phylotype richness (i.e., number of rarefied OTUs) indices were calculated by the QIIME toolkit, with rarefaction analysis of 250 bootstrap random sampling iterations and 4% incremental sampling efforts. For β-diversity analysis, dissimilarities of the microeukaryotic communities were calculated using principal coordinate analysis (PCoA) of normalized, weighted, pairwise UniFrac (Lozupone and Knight, 2005) distances between all samples, of which principal component eigenvalues were generated by the QIIME toolkit. Analysis of similarity (ANOSIM) based on 999 permutations was performed using the Bray-Curtis (Bray and Curtis, 1957) algorithm to quantitatively compare the community differences between different groups. Redundancy analysis (RDA) related microbial properties to the explanation of the fumigated communities. In addition, Mantel test revealed the correlations between microbial properties and the community composition of total microeukaryotes, fungi and protists in the fumigated soils. These analyses were completed in the package “vegan” of the R project (version 3.1.3) (http://www.r-project.org/). A heat map was constructed using the function “heatmap.2” from the R package “gplots.” For better visualization, the original data were transformed following the formula log_2_ (1000*x* + 1) (Lundberg et al., 2012), and hierarchical clustering was based on Bray-Curtis similarities with group-average linkage. A Venn diagram was employed to characterize the shared and unique microeukaryotic communities among different treatments. One-way ANOVA was performed using SPSS 16.0 software, and significant differences were determined using Bonferroni's multiple range test.

Microeukaryotic co-occurrence networks were constructed using the online CoNet pipeline (http://apps.cytoscape.org/apps/conet) to explore the internal community relationships. OTUs with less than 10 sequences were filtered to remove poorly represented OTUs and reduce network complexity (Barberán et al., 2012). All possible Spearman's rank correlations between OTUs were calculated. The valid co-occurrence patterns were considered with the Spearman's correlation coefficient *r* > 0.6 and significance *P* < 0.01 (Barberán et al., 2012). The nodes in the network represent the OTUs at 97% identity, and the connections correspond to a strong and significant correlation between nodes. The topological properties (i.e., average path length, cumulative degree distribution, network diameter, clustering coefficient, modularity, eccentricity, closeness, and betweenness centrality) were calculated in the platform Gephi (Bastian et al., 2009). Visualization of the network was also performed in the Gephi.

### Nucleotide sequence deposition

All sequencing datasets were deposited in the National Center for Biotechnology Information (NCBI) Sequence Read Archive (http://trace.ncbi.nlm.nih.gov/Traces/sra/) under study SRP058996 with BioSample accessions SAMN03751795–SAMN03751830.

## Results

### Microbial biomass and activities

After fumigation, the amount of microbial biomass C significantly decreased by approximately 70% in the grassland and arable soils during the incubation period, and it was significantly lower at day 30 compared to day 0 (Table 2). Both fumigated soils at day 0 showed a significantly higher rate of microbial respiration than other treatments during the incubation period. The change in β-glucosidase activity in both soils was not significant during the incubation period. Both fumigated soils at days 0 and 7 had significantly higher invertase activities than other treatments. Fumigation enhanced the metabolic quotient in both soils at day 30. Specific β-glucosidase activity in the grassland and arable soils was increased by fumigation by average 4.5 and 4.4-fold, respectively, and specific invertase activity by average 5.7 and 8.2-fold, respectively (Table 2).

**Table 2:** **Microbial properties in different treatments during the incubation period**.

	**Microbial biomass C (μg g^−1^)**	**Microbial respiration rate (μg CO_2_ g^−1^ h^−1^)**	**β-glucosidase activity (μg PNP g^−1^ h^−1^)**	**Invertase activity (μg glucose g^−1^ h^−1^)**	**Metabolic quotient^c^ (μg CO_2_ μg^−1^ MBC h^−1^)**	**Specific β-glucosidase/invertase activity^d^**	
						**(μg PNP μg^−1^ MBC h^−1^)**	**(μg glucose μg^−1^ MBC h^−1^)**
G-F-0^a^	72.43 (11.15) b^b^	3.68 (0.37) a	285.61 (21.97) a	1652.97 (108.84) a	0.05 (0.01) ab	3.98 (0.34) b	23.37 (5.42) ab
G-F-7	55.20 (13.36) bc	1.70 (0.20) b	270.00 (21.89) a	1451.02 (80.20) a	0.03 (0.01) ab	5.02 (0.82) ab	27.03 (4.65) a
G-F-30	24.99 (11.79) c	1.68 (0.22) b	271.54 (11.45) a	831.45 (92.61) b	0.08 (0.05) a	12.79 (6.48) a	37.82 (15.00) a
G-0	160.86 (1.67) a	1.66 (0.06) b	255.26 (12.19) a	865.68 (37.03) b	0.02 (0.00) b	1.60 (0.18) b	5.42 (0.53) b
G-7	174.87 (11.39) a	1.65 (0.07) b	263.62 (15.60) a	838.30 (15.69) b	0.01 (0.00) b	1.57 (0.45) b	4.96 (1.22) b
G-30	156.66 (15.07) a	1.65 (0.06) b	254.82 (8.96) a	797.22 (58.39) b	0.01 (0.00) b	1.63 (0.10) b	5.12 (0.58) b
A-F-0	68.88 (12.35) b	2.28 (0.45) a	167.47 (43.57) a	2071.05 (44.78) a	0.03 (0.01) ab	2.54 (1.05) a	30.67 (5.19) ab
A-F-7	37.67 (10.93) bc	1.13 (0.02) b	145.21 (14.20) a	2341.10 (184.19) a	0.03 (0.01) ab	4.05 (1.01) a	66.85 (25.11) a
A-F-30	29.80 (14.66) c	1.11 (0.03) b	133.16 (13.48) a	791.72 (78.22) b	0.05 (0.03) a	5.59 (3.58) a	33.10 (20.95) ab
A-0	147.56 (8.77) a	1.11 (0.02) b	144.42 (5.76) a	841.72 (38.88) b	0.01 (0.00) b	0.98 (0.14) a	5.72 (0.49) b
A-7	162.70 (5.00) a	1.08 (0.01) b	144.76 (11.55) a	769.84 (97.60) b	0.01 (0.00) b	0.91 (0.15) a	4.89 (1.18) b
A-30	141.41 (11.38) a	1.09 (0.06) b	127.58 (13.98) a	742.45 (27.17) b	0.01 (0.01) b	0.91 (0.17) a	5.27 (0.48) b

a G, grassland soil; A, arable soil; F, fumigation; number, incubation days.

b Standard deviations of three replicates in parentheses. Different letters indicate significant differences at P < 0.05.

c Microbial respiration rate divided by microbial biomass C.

d The respective enzyme activity per unit biomass C.

### Taxonomic assemblages of microeukaryotes

**Figure 1:**
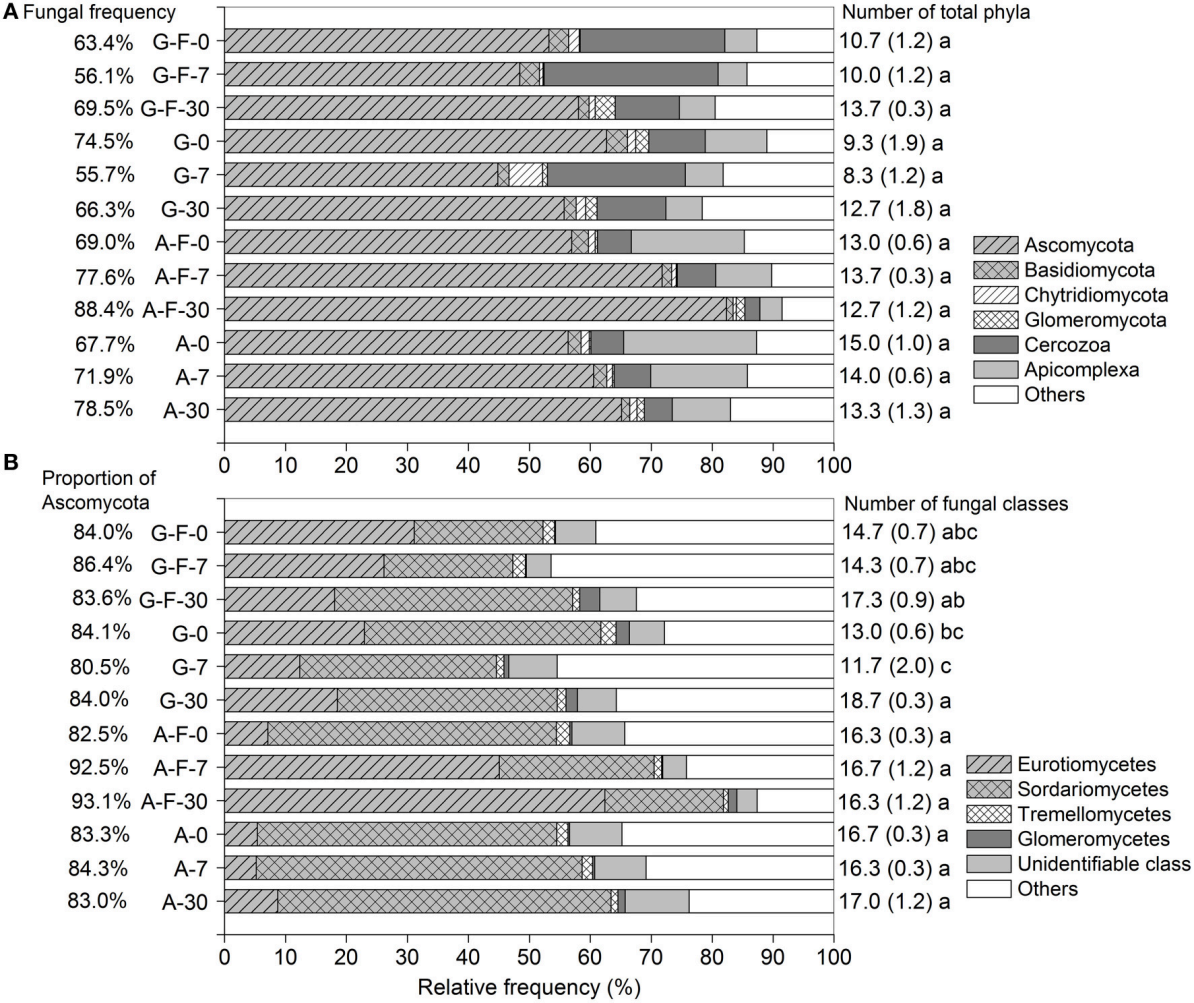
**Taxonomic distributions of the microeukaryotic phyla (A) and the fungal classes (B) in different treatments during the incubation period**. Each stripe represents the average frequency of three replicates. Percentages in panels **(A,B)** are the fungal frequency and the proportion of Ascomycota in the fungal community, respectively. Numbers on the right are the counts for total phyla **(A)** and fungal classes **(B)**, with the respective standard deviation in parentheses, and the same letter indicates no significant difference within the grassland or arable treatment. G, grassland soil; A, arable soil; F, fumigation; number, incubation days.

Across all soil samples, a total of 474,982 high-quality sequences (99.9% were retrieved from eukaryota), clustered into 6664 OTUs after trimming and filtration (Table S1). The microeukaryotic communities were dominated by fungi, which accounted for 55.7–88.4% of the total sequences among different treatments. Ascomycota, Cercozoa, and Apicomplexa (belonging to fungi, Rhizaria, and Alveolata, respectively) were the major phyla. These phyla in the fumigated grassland soil showed no consistent changes during the incubation period, while in the fumigated arable soil, the relative frequency of Ascomycota (especially the class Eurotiomycetes, Figure 1B) increased while that of Apicomplexa decreased with increasing incubation time (Figure 1A). The fungal community was dominated by Ascomycota (mainly the subphylum Pezizomycotina, Table S2) (80.5–93.1% of the fungal sequences), in which the classes Eurotiomycetes and Sordariomycetes showed high abundance (Figure 1B). Overall, fumigation modulated taxonomic composition of the microeukaryotic communities at the phylum/class levels. Especially in the arable soil, the predominance of the class Sordariomycetes was replaced by the class Eurotiomycetes after fumigation at days 7 and 30 (Figure 1B). During the incubation period, there were no significant differences in the number of total phyla in the grassland or arable treatment (Figure 1A), and the number of fungal classes in the arable treatment (Figure 1B).

### OTU distribution and network analysis

We used a Venn diagram to observe shared and unique communities among different treatments at the end of incubation (Figure S1). The Venn diagram was constructed based on a subset of 5000 sequences per sample and the average OTUs based on three replicates. The fumigated grassland and arable soils harbored 26 and 19 unique OTUs, respectively (accounting for 18.2 and 16.0% of the respective total OTUs), and they shared 96 and 76 OTUs with their corresponding non-fumigated controls, in which 39 and 38 were unique OTUs. Both fumigated soils exclusively shared 5 OTUs, only accounting for 3.5 and 4.2% of the respective communities. The 59 common OTUs were shared by all treatments (Figure S1).

The co-occurrence patterns in the fumigated microeukaryotic communities through 30-day incubation were explored by construction of OTU networks (Figures S2, S3). The fumigated communities exhibited 190 significant correlations (connections) of 74 OTUs (nodes) in the grassland soil (Figure S2), and 192 significant correlations of 88 OTUs in the arable soil (Figure S3). The fungal OTUs were shown to be important nodes closely related to other OTUs, and accounted for 56.8 and 51.1% of nodes in the fumigated grassland and arable soils, respectively. The average path lengths were 1.73 and 2.02 in the networks of the fumigated grassland and arable soils, respectively, and network diameters were both 5. These topological properties indicated that the microeukaryotic communities in both fumigated soils were highly connected and presented small-world networks (short network distance among most of nodes and their interconnections through several paths).

### Microeukaryotic α- and β-diversity

**Figure 2:**
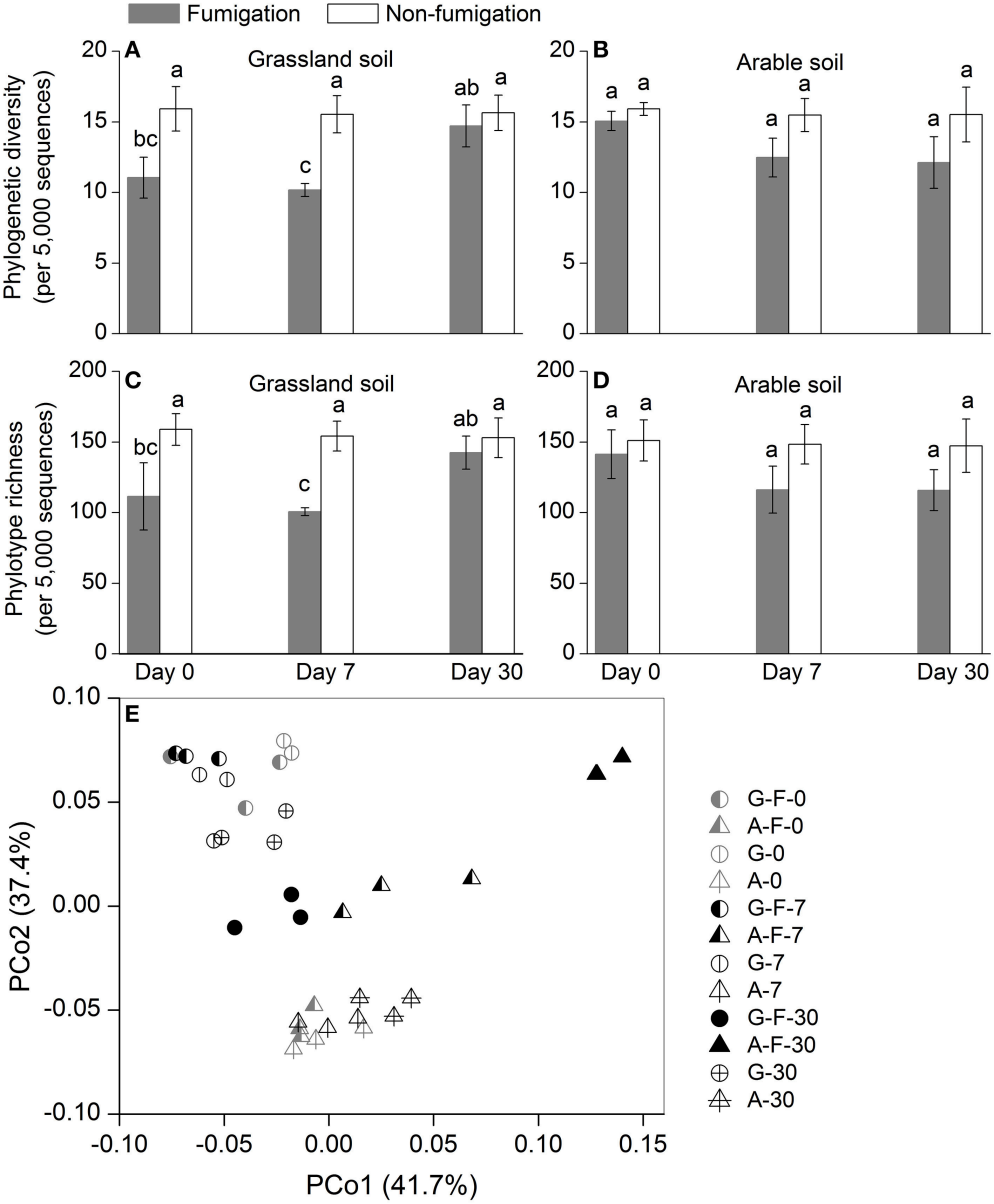
**Microeukaryotic α- and β-diversity in different treatments during the incubation period**. Phylogenetic diversity **(A, B)** and phylotype richness **(C, D)** were calculated based on rarefaction to 5000 sequences, the bars indicate ±1 standard deviations of three replicates, and different letters indicate significant differences at *P* < 0.05. Microeukaryotic community structure was indicated by principal coordinate analysis (PCoA) of the normalized and weighted pairwise UniFrac distances between all samples **(E)**. G, grassland soil; A, arable soil; F, fumigation; number, incubation days.

Phylogenetic diversity and phylotype richness indices based on rarefaction to 5000 sequences were used to estimate the microeukaryotic α-diversity (Figures 2A–D). After fumigation, the grassland soil microeukaryotic α-diversity at days 0 and 7 significantly decreased, compared with the corresponding non-fumigated controls (Figures 2A,C). Compared with the grassland soil, the arable soil exhibited different response of the microeukaryotic α-diversity to fumigation. During the incubation period, the microeukaryotic α-diversity between the fumigated and non-fumigated arable soil showed no statistical difference (Figures 2B,D).

The profiles of the microeukaryotic community structure were plotted using PCoA of the normalized and weighted pairwise UniFrac distances between all samples (Figure 2E). The fumigated microeukaryotic communities at the start of the incubation were not separated from the corresponding non-fumigated communities, which clustered well together during the incubation period. In the fumigated grassland soil, the microeukaryotic communities at day 30 were moderately separated from those at days 0 and 7. In the fumigated arable soil, the visible differentiations of community structure between different incubation time points occurred along the second coordinate axis (PCo2). The separation of the microeukaryotic communities in the first component (PCo1) implied that the two soils had different microeukaryotic community structures. The results of ANOSIM (Table 3) further confirmed the significant (*P* < 0.01) effects of fumigation and soil source on the microeukaryotic community structure. Fumigation had no statistical effect at day 0 but significant (*P* < 0.05) effect at days 7 and 30 on community structure in the arable soil (Table 3, Figure 2E).

**Table 3:** **Effects of fumigation, soil, and incubation time on the microeukaryotic community structure**.

**Source**	**R**	***P***	**Time**	**R**	***P***
Fumigation	0.205	0.005	Day 0	0.154	0.081
			Day 7	0.285	0.030
			Day 30	0.304	0.018
Soil	0.406	0.001	Day 0	0.248	0.022
			Day 7	0.367	0.012
			Day 30	0.480	0.003
Time	0.094	0.053			

Statistic R and significance (P) of differences between different groups (fumigation and non-fumigation, grassland and arable soils, and different incubation time) were calculated by analysis of similarity (ANOSIM) using OTU-based Bray–Curtis distances.

### The main phylotypes in response to fumigation

**Figure 3:**
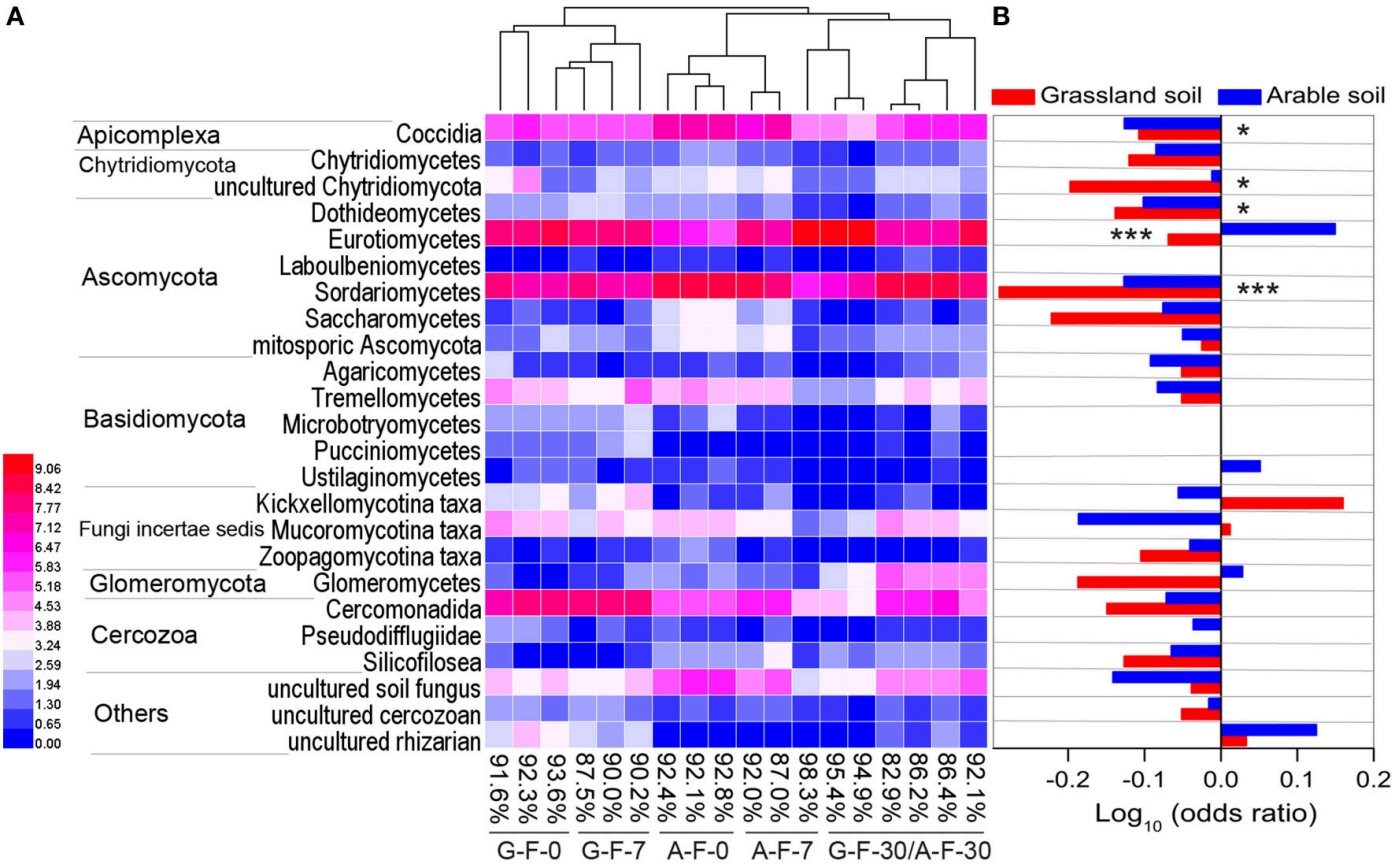
**The main microeukaryotic phylotypes and their responses to fumigation**. Heat map **(A)** showing the visualized distributions of the main classes in both fumigated soils during the incubation period. Values were transformed following the formula log_2_ (1000*x* + 1), where *x* is the frequency of individual taxon, and percentages are the total frequencies of the chosen taxa in the corresponding sample communities. The base-10 logarithm of the odds ratio **(B)**: the ratio of the odds of a taxon occurring in the fumigated treatment to the odds of it occurring in the non-fumigated treatment. ^*^ and ^***^ mark significance at *P* < 0.05 and 0.001, respectively, based on independent-samples *T*-test of Blom-normalized frequencies. G, grassland soil; A, arable soil; F, fumigation; number, incubation days.

The microeukaryotic classes in which the relative frequencies exceeded 0.1% were selected to construct a heat map of distributions of the main microeukaryotes after fumigation (Figure 3A). Figure 3B showed the log_10_-transformed odds ratio, which is the ratio of the odds of a given phylotype occurring in the fumigated soils to the odds of it occurring in the corresponding non-fumigated controls, based on OTU counts pooled across incubation days. The selected phylotypes made up 82.9–98.3% (91.0 ± 3.8%) of the total sequences in the fumigated samples (Figure 3A). Hierarchical clustering demonstrated that the fumigated treatments at day 30 clustered better than those at days 0 and 7. After fumigation, the classes Eurotiomycetes and Sordariomycetes were the core phylotypes (Figures 1B, 3A), with average 21.9 and 36.5% relative frequency, respectively (Table S3).

In the two soils, only 3–4 phylotypes (uncultured rhizarian, Kickxellomycotina, and Mucoromycotina taxa for the grassland soil; Eurotiomycetes, Ustilaginomycetes, Glomeromycetes, and uncultured rhizarian for the arable soil) were positively stimulated by fumigation. Most other phylotypes were more likely to inhabit the non-fumigated soils. Notably, Sordariomycetes, Dothideomycetes, Coccidia, and uncultured Chytridiomycota were significantly inhibited by fumigation. Some phylotypes (e.g., Eurotiomycetes, Glomeromycetes, and Kickxellomycotina taxa) in the two soils showed different responses to fumigation (Figure 3B).

### Relationships between microbial properties and the fumigated microeukaryotic communities

**Figure 4:**
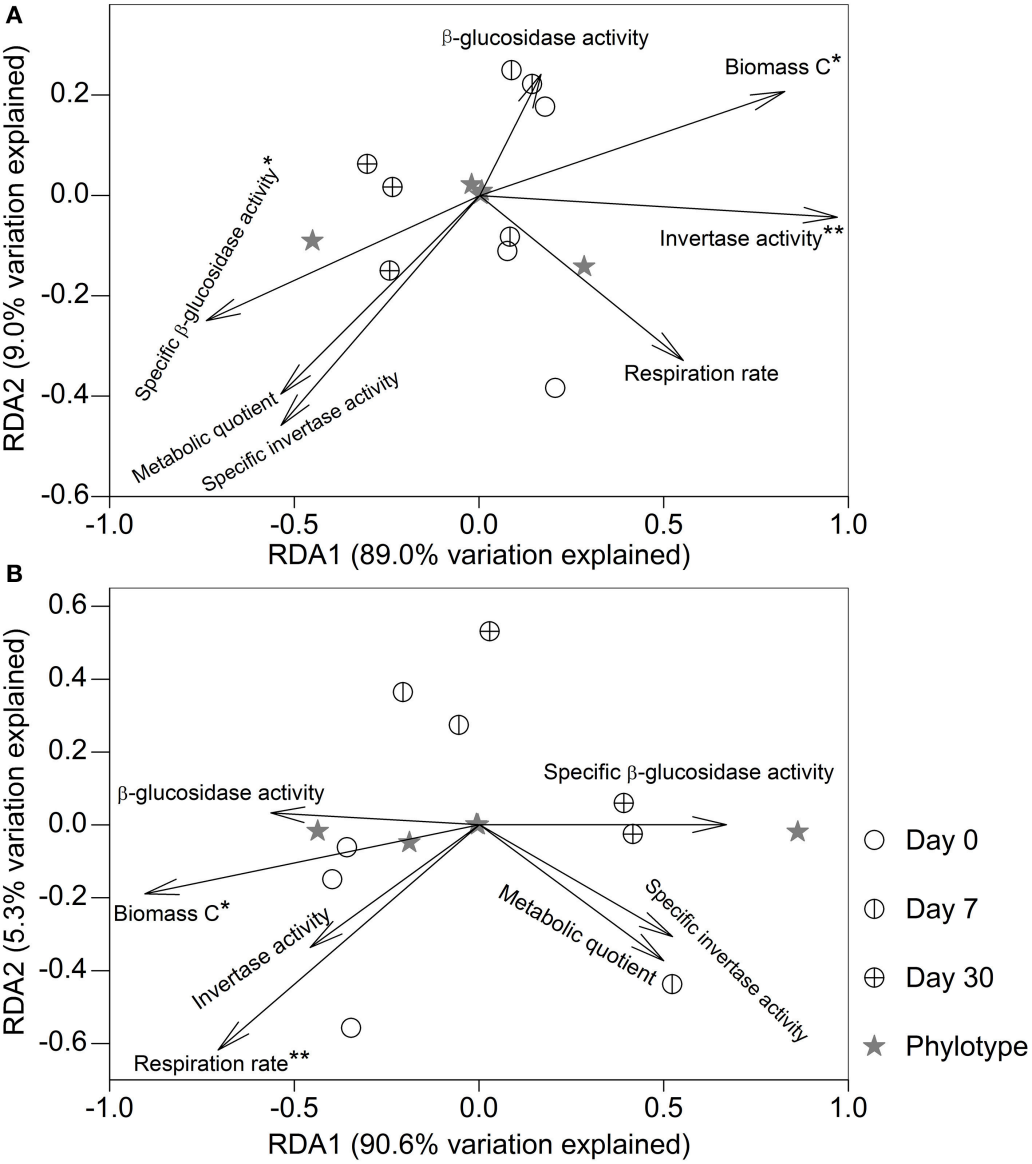
**Redundancy analysis relating microbial properties to the main microeukaryotic sequence patterns after fumigation**. Panels **(A,B)** indicate the fumigated grassland and arable soils, respectively. The length of each arrow indicates the contribution of the corresponding parameter to the structural variation. ^*^ and ^**^ mark significance at *P* < 0.05 and 0.01, respectively, based on 999 Monte Carlo permutations.

After fumigation, irrespective of incubation time, the significantly varied microeukaryotic phylotypes (i.e., Eurotiomycetes, Sordariomycetes, Dothideomycetes, Coccidia, and uncultured Chytridiomycota, Figure 3B) in the two soils were used to relate microbial properties. RDA indicated that the variation in these microeukaryotic phylotypes was significantly explained by invertase activity, specific β-glucosidase activity, and biomass C in the fumigated grassland soil (Figure 4A), and by respiration rate and biomass C in the fumigated arable soil (Figure 4B). The Mantel test revealed that invertase activity, specific β-glucosidase activity, and biomass C were significantly correlated with the community composition of total microeukaryotes, fungi, and protists in the fumigated grassland soil (Table 4).

**Table 4:** **Mantel test showing the correlations between microbial properties and the community composition of total microeukaryotes, fungi, and protists in the fumigated soils**.

	**Variable**	**Total microeukaryotes**		**Fungi**		**Protists**	
		***r***	***P***	***r***	***P***	***r***	***P***
Fumigated grassland soil	Microbial biomass C	**0.56**	**0.02**	**0.56**	**0.01**	**0.49**	**0.02**
	Microbial respiration rate	0.09	0.26	0.06	0.31	−0.02	0.47
	β-glucosidase activity	−0.12	0.74	−0.08	0.61	−0.16	0.87
	Invertase activity	**0.88**	<**0.01**	**0.88**	<**0.01**	**0.77**	**0.01**
	Metabolic quotient	0.24	0.10	0.15	0.17	**0.43**	**0.02**
	Specific β-glucosidase activity	**0.44**	**0.01**	**0.38**	**0.04**	**0.58**	**0.01**
	Specific invertase activity	0.19	0.17	0.13	0.22	0.36	0.07
Fumigated arable soil	Microbial biomass C	**0.65**	**0.01**	**0.69**	<**0.01**	**0.47**	**0.01**
	Microbial respiration rate	**0.29**	**0.05**	**0.33**	**0.04**	0.09	0.18
	β-glucosidase activity	0.06	0.29	0.08	0.26	−0.02	0.51
	Invertase activity	0.28	0.08	0.23	0.10	**0.44**	**0.02**
	Metabolic quotient	0.11	0.19	0.11	0.22	0.19	0.15
	Specific β-glucosidase activity	**0.28**	**0.04**	**0.29**	**0.02**	0.28	0.07
	Specific invertase activity	0.22	0.07	0.23	0.06	0.15	0.19

The bold values indicate the significant (P < 0.05) correlations and their corresponding Spearman's rank coefficients (r).

## Discussion

### Detailed information on the grassland and arable soil microeukaryotic communities

The present study is the first work to extensively investigate the microeukaryotic communities in the fumigated soils by means of deep MiSeq sequencing of the eukaryotic 18S rRNA gene amplicons. A total of 474,982 quality filtered reads were clustered into 6664 OTUs across 36 sample datasets. These data provided detailed information on taxonomic composition and diversity patterns of the grassland and arable soil microeukaryotic communities, and further revealed their temporal evolution in response to fumigation. Compared with other studies in terms of soil eukaryotic microbiota, we obtained the more numerous sequences which were rarefied to a deeper level (5000 sequences per sample) for diversity analysis. For example, Chen et al. (2012) only measured 793 gene sequences for microeukaryotic community analysis in a continuous peanut-cropping area. In the studies of Shen et al. (2014) and Shi et al. (2015), the soil microeukaryotic datasets were only rarefied to approximately 1000 sequences per sample for diversity analysis. In addition, our information extends current knowledge of the grassland and arable soil microeukaryotic communities, which are derived from analyses of traditional genetic fingerprinting, clone library, and culture-dependent assays (e.g., Marschner et al., 2003; Moon-van der Staay et al., 2006; Lara et al., 2007; Tzeneva et al., 2009).

In grassland ecosystems, the soil microeukaryotic community composition, and diversity are strongly influenced by above-ground vegetation structure (e.g., plant height, species diversity and richness, functional type, and composition) (Sugiyama et al., 2008; Prober et al., 2015). The arable soil microeukaryotic communities are affected by different agricultural management practices. In a long-term fertilization experiment, Lentendu et al. (2014) observed that the eukaryotic datasets were dominated by Streptophyta sequences, followed by fungal and microfauna sequences. The changes in soil pH, moisture and nutrient availability caused by fertilization affected the microeukaryotic community composition in the arable soil (Lentendu et al., 2014). In our study, the grassland and arable soil microeukaryotic communities were dominated by fungi, accounting for 55.7–88.4% of the total eukaryotic sequences. The majority of fungal sequences belonged to the phylum Ascomycota (Figure 1, Table S2), which is usual for soil habitats lacking ectomycorrhizal host plants (Schadt et al., 2003). Similar findings are observed in other grassland and arable soils using clone library constructing and molecular genetic fingerprinting based on the biomarker of fungal internal transcribed spacer (ITS) region gene (de Castro et al., 2008; Klaubauf et al., 2010; Karst et al., 2013). Previous results indicated that Ascomycota dominated the fungal community in a maize-wheat rotation soil during the process of straw decomposition (Chen et al., 2014). The majority of Ascomycota belong to the fast-growing fungal populations (or *r*-strategists) which preferentially metabolize easily degradable fractions of organic matter (Lundell et al., 2010), and are abundant in soils with relatively high N contents (Nemergut et al., 2008). However, other studies suggest that many Ascomycota groups have distinctive morphological features that confer extensive stress tolerance and permit survival in hostile environments (e.g., Sterflinger et al., 2012; Nai et al., 2013). These reports support our findings that Ascomycota similarly dominated the microeukaryotic communities in the fumigated soil (Figure 1A). Actually, the phyla Ascomycota and Basidiomycota represent the main classified fungal decomposers in soils (Vandenkoornhuyse et al., 2002). The grassland soil analyzed by Anderson et al. (2003) was well colonized by Basidiomycota [60% of the clones in the combined small sub-unit (SSU) library and 47% in the ITS library], while their abundance was relatively low in our study (Figure 1A, Table S2). By conducting a long-term elevated CO_2_ (eCO_2_) experiment on a secondary successional grassland (aCO_2_ as control), Tu et al. (2015) observed that the fungal community was dominated by Ascomycota (77 and 81% of the fungal sequences for eCO_2_ and aCO_2_, respectively), followed by Basidiomycota. In addition, two protist groups (i.e., Cercozoa and Apicomplexa) were moderately abundant in the grassland and arable soil microeukaryotic communities (Figure 1A). In several German grassland soils, Domonell et al. (2013) found Cercozoa (abundance 32.4 ± 13.2%) as one of the dominant protists. Other studies also observed the existence of Cercozoa and Apicomplexa with moderate abundance in typical Chinese soils (Jing et al., 2014; Shen et al., 2014; Shi et al., 2015).

### Composition, diversity, and C-metabolic functions of the microeukaryotic communities in response to short-term fumigation-incubation

Overall, the number of total microeukaryotic phyla and fungal classes were not greatly decreased by fumigation during the incubation period (Figures 1A,B). Fumigation showed no significant effect on the observed OTU richness at the end of incubation (Figure S1; Figures 2C,D). Therefore, fumigation does not sharply reduce the soil microeukaryotic taxa, but rather biomass size during short-term fumigation-incubation (Zelles et al., 1997; Dickens and Anderson, 1999). Taxonomic composition of the microeukaryotic communities at the phylum/class levels was modulated by fumigation (Figures 1A,B). Fumigation significantly decreased the grassland soil microeukaryotic diversity and richness at days 0 and 7, and changed the arable soil community structure at days 7 and 30 (Figures 2A,C,E, Table 3). After fumigation, the successions of the arable soil microeukaryotic communities occurred with soil incubation (Figure 2E). Previous studies indicated that after fumigation the surviving microorganisms mineralized the necromass released from cell lyses within several days (Jenkinson and Powlson, 1976; Wu et al., 1996; Kemmitt et al., 2008). As nutrient conditions change (i.e., following fumigant removal there is a release of necromass), the redistribution of the microeukaryotic communities (i.e., copiotrophs and oligotrophs) probably occurs, leading to the changed community structure during the incubation period. Similarly, in a straw amendment incubation experiment, we also observed the redistributions of the arable soil bacterial and microeukaryotic communities as straw availability declined over time (Chen et al., 2014, 2015).

The main microeukaryotic phylotypes in response to fumigation were reflected by the base-10 logarithm of the odds ratio (Ganesh et al., 2014). Positive values indicate taxa that are more likely to occur in the fumigated treatments. Most phylotypes were inhibited by fumigation, only 3 and 4 phylotypes in the grassland and arable soils respectively were positively stimulated by fumigation. In the arable soil, fumigation showed a very significant stimulation of Eurotiomycetes (Figure 3B). Many Eurotiomycetes species are adaptable and resilient in extreme ecosystems (e.g., heat, drought, oligotrophy, and hypersalinity) (Kis-Papo et al., 2001; Sterflinger et al., 2012; Nai et al., 2013). These abilities can facilitate their recolonization in acidic arable soils after fumigation. Eurotiomycetes, Glomeromycetes, Kickxellomycotina, and Mucoromycotina taxa in the two soils showed different responses to fumigation (Figures 2, 3B, Table 3). This can be ascribed to the distinctly different habitat conditions in the two soils. The grassland and arable soils contained 26.6 and 15.8 g kg^−1^ organic C respectively, with soil pHs of 7.1 and 4.4 (Table 1).

In soil microbiology, there is a “paradox” of SOC mineralization, which is that even though majority of the soil microorganisms are killed by fumigation, SOC mineralization continues at the same rate as in the non-fumigated soil for several weeks or even months (Jenkinson and Powlson, 1976; Wu et al., 1996; Kemmitt et al., 2008). This phenomenon can be partly explained by the microeukaryotes that survive fumigation (mainly the phylotypes Eurotiomycetes and Sordariomycetes, Figure 4A), due to their metabolic functions in C turnover and energy flow (Chen et al., 2012, 2014; Damon et al., 2012; Jing et al., 2014). For instance, Eurotiomycetes belong to cellulolytic fungi and can produce extracellular cellulases based on fungal cellobiohydrolase (*cbhl*) gene characterization (Fan et al., 2012). The Sordariomycetes species are capable of decomposing the organic residues in soils, attributed to the excretion of carboxylases and amidolyases (Strope et al., 2011).

Soil β-glucosidase and invertase activities are two useful indicators involved in the decomposition of organic C (Nannipieri et al., 2012). A flush of invertase activity occurred following fumigation (Table 2), as some intracellular enzymes were released into the soils during cell lyses. Some enzymes released during cell lyses can resist proteolysis and maintain their activities during and after fumigation (Renella et al., 2002). Specific enzyme activity, an activity index of microbial biomass, can be expressed as soil enzyme activity per unit biomass C (Waldrop et al., 2000). Specific β-glucosidase activity was greater in the fumigated soils compared to the non-fumigated soils (Table 2), but those changes were not significant. In the fumigated grassland soil, the variation in the main microeukaryotes was significantly explained by invertase activity, biomass C and specific β-glucosidase activity, and they were well correlated with the community composition of total microeukaryotes, fungi, and protists (Figure 4A, Table 4). The co-occurrence networks indicated that after fumigation the internal communities in the grassland and arable soils were highly connected, mainly the connections of fungi to other taxa (Figures S2, S3), mirroring the diverse linkages between the microeukaryotic groups in terms of their ecological functions. Therefore, the fumigated microeukaryotic communities probably make a large contribution to SOC mineralization by release of hydrolytic enzymes and their activities.

Our study is also supported by two basic principles in soil microbiology. Firstly, soil is considered to have a large excess pool of total microbial biomass, whereas only a small portion of the microbial biomass is active (excessive pool principle) (Morris and Blackwood, 2007). Secondly, many similar functions can be carried out by different microbial taxonomic groups (redundancy principle) (Stres and Tiedje, 2006). In our study, despite the greatly decreased biomass C after fumigation, the residual fraction of microeukaryotes that survive fumigation are still active to drive SOC mineralization. Different microeukaryotic phylotypes (e.g., Eurotiomycetes and Sordariomycetes) have similar functions in SOC mineralization by producing a variety of hydrolytic enzymes.

Collectively, combined with previous studies based on PLFA analysis (Zelles et al., 1997; Dickens and Anderson, 1999), our study showed that short-term fumigation-incubation not only reduced the biomass size of the microeukaryotic communities, but changed their α-diversity in the grassland soil, β-diversity in the arable soil, and taxonomic composition in both soils. The co-occurrence networks indicated that after fumigation the internal microeukaryotic communities were highly connected, mainly the connections of fungi to other taxa. The fumigated microeukaryotic communities retain the ability to drive SOC mineralization by release of hydrolytic enzymes and their activities, despite the greatly decreased microeukaryotic biomass.

### Conflict of interest statement

The authors declare that the research was conducted in the absence of any commercial or financial relationships that could be construed as a potential conflict of interest.

## Supplementary material

Figure S1 **Venn diagram illuminating the amounts of shared and unique OTUs among different treatments at day 30 of the incubation**. OTUs were counted from a subset of 5000 sequences per sample, and the average OTUs of three replicates were enumerated.

Figure S2 **Network analysis on the microeukaryotic communities in the fumigated grassland soil through 30-day incubation**. Colored nodes were the OTUs at 97% identity, and the connections indicate significant correlations (*r* > 0.6, *P* < 0.01). The size of each node is proportional to the number of connections (degree).

Figure S3 **Network analysis on the microeukaryotic communities in the fumigated arable soil through 30-day incubation**. Colored nodes were the OTUs at 97% identity, and the connections indicate significant correlations (*r* > 0.6, *P* < 0.01). The size of each node is proportional to the number of connections (degree).

Table S1 **Number of sequences and OTUs measured by QIIME processing**.

Table S2 **Relative frequency (%) of the main subphyla affiliated with Ascomycota and Basidiomycota**.

Table S3 **Relative frequency (%) of the main identifiable taxa**.

## References

[B1] Anderson I. C. Campbell C. D. Prosser J. I. (2003). Potential bias of fungal 18S rDNA and internal transcribed spacer polymerase chain reaction primers for estimating fungal biodiversity in soil. Environ. Microbiol. 5, 36–47. 10.1046/j.1462-2920.2003.00383.x 12542711

[B2] Bandick A. K. Dick R. P. (1999). Field management effects on soil enzyme activities. Soil Biol. Biochem. 31, 1471–1479. 10.1016/S0038-0717(99)00051-6

[B3] Barberán A. Bates S. T. Casamayor E. O. Fierer N. (2012). Using network analysis to explore co-occurrence patterns in soil microbial communities. ISME J. 6, 343–351. 10.1038/ismej.2011.119 21900968 PMC3260507

[B4] Bastian M. Heymann S. Jacomy M. (2009). Gephi: an open source software for exploring and manipulating networks, in International AAAI Conference on Weblogs and Social Media (San Jose, CA).

[B5] Blagodatskaya E. V. Anderson T. H. (1998). Interactive effects of pH and substrate quality on the fungal-to-bacterial ratio and qCO2 of microbial communities in forest soils. Soil Biol. Biochem. 30, 1269–1274. 10.1016/S0038-0717(98)00050-9

[B6] Bray J. R. Curtis J. T. (1957). An ordination of the upland forest communities of Southern Wisconsin. Ecol. Monogr. 27, 326–349. 10.2307/1942268

[B7] Bremner J. M. (1965). Total nitrogen, in Chemical and Microbiological Properties, No. 9, Agronomy, eds Black C. A. Evans D. D. Ensminger L. E. White J. L. Clark F. E. Dinauer R. C. (Madison, WI: American Society of Agronomy), 1149–1178.

[B8] Brookes P. C. Kemmitt S. J. Addiscott T. M. Bird N. (2009). Reply to Kuzyakov et al.'s comments on our paper: ‘Kemmitt, S. J., Lanyon, C. V., Waite, I. S., Wen, Q., O'Donnell, A. G., Brookes, P. C., 2008. Mineralization of native soil organic matter is not regulated by the size, activity or composition of the soil microbial biomass–a new perspective. Soil Biology & Biochemistry 40, 61–73'. Soil Biol. Biochem. 41, 440–443. 10.1016/j.soilbio.2008.09.002

[B9] Brookes P. C. Landman A. Pruden G. Jenkinson D. S. (1985). Chloroform fumigation and the release of soil-nitrogen–a rapid direct extraction method to measure microbial biomass nitrogen in soil. Soil Biol. Biochem. 17, 837–842. 10.1016/0038-0717(85)90144-0

[B10] Brookes P. C. Powlson D. S. Jenkinson D. S. (1982). Measurement of microbial biomass phosphorus in soil. Soil Biol. Biochem. 14, 319–329. 10.1016/0038-0717(82)90001-3

[B11] Caporaso J. G. Bittinger K. Bushman F. D. DeSantis T. Z. Andersen G. L. Knight R. (2010b). PyNAST: a flexible tool for aligning sequences to a template alignment. Bioinformatics 26, 266–267. 10.1093/bioinformatics/btp636 19914921 PMC2804299

[B12] Caporaso J. G. Kuczynski J. Stombaugh J. Bittinger K. Bushman F. D. Costello E. K.. (2010a). QIIME allows analysis of high-throughput community sequencing data. Nat. Methods 7, 335–336. 10.1038/nmeth.f.303 20383131 PMC3156573

[B13] Chen L. Zhang J. Zhao B. Yan P. Zhou G. Xin X. (2014). Effects of straw amendment and moisture on microbial communities in Chinese fluvo-aquic soil. J. Soil Sediment 14, 1829–1840. 10.1007/s11368-014-0924-2

[B14] Chen L. Zhang J. Zhao B. Zhou G. Ruan L. (2015). Bacterial community structure in maize stubble-amended soils with different moisture levels estimated by bar-coded pyrosequencing. Appl. Soil Ecol. 86, 62–70. 10.1016/j.apsoil.2014.09.011

[B15] Chen M. Li X. Yang Q. Chi X. Pan L. Chen N.. (2012). Soil eukaryotic microorganism succession as affected by continuous cropping of peanut–pathogenic and beneficial fungi were selected. PLoS ONE 7:e40659. 10.1371/journal.pone.0040659 22808226 PMC3393692

[B16] Damon C. Lehembre F. Oger-Desfeux C. Luis P. Ranger J. Fraissinet-Tachet L.. (2012). Metatranscriptomics reveals the diversity of genes expressed by eukaryotes in forest soils. PLoS ONE 7:e28967. 10.1371/journal.pone.0028967 22238585 PMC3253082

[B17] de Castro A. P. Quirino B. F. Pappas G. Jr. Kurokawa A. S. Neto E. L. Krüeger R. H. (2008). Diversity of soil fungal communities of Cerrado and its closely surrounding agriculture fields. Arch. Microbiol. 190, 129–139. 10.1007/s00203-008-0374-6 18458875

[B18] Dickens H. E. Anderson J. M. (1999). Manipulation of soil microbial community structure in bog and forest soils using chloroform fumigation. Soil Biol. Biochem. 31, 2049–2058. 10.1016/S0038-0717(99)00128-5

[B19] Dominguez-Mendoza C. A. Bello-Lopez J. M. Navarro-Noya Y. E. de Leon-Lorenzana A. S. Delgado-Balbuena L. Gomez-Acata S. (2014). Bacterial community structure in fumigated soil. Soil Biol. Biochem. 73, 122–129. 10.1016/j.soilbio.2014.02.012

[B20] Domonell A. Brabender M. Nitsche F. Bonkowski M. Arndt H. (2013). Community structure of cultivable protists in different grassland and forest soils of Thuringia. Pedobiologia 56, 1–7. 10.1016/j.pedobi.2012.07.001

[B21] Edgar R. C. (2010). Search and clustering orders of magnitude faster than BLAST. Bioinformatics 26, 2460–2461. 10.1093/bioinformatics/btq461 20709691

[B22] Edgar R. C. Haas B. J. Clemente J. C. Quince C. Knight R. (2011). UCHIME improves sensitivity and speed of chimera detection. Bioinformatics 27, 2194–2200. 10.1093/bioinformatics/btr381 21700674 PMC3150044

[B23] Fan F. Li Z. Wakelin S. A. Yu W. Liang Y. (2012). Mineral fertilizer alters cellulolytic community structure and suppresses soil cellobiohydrolase activity in a long-term fertilization experiment. Soil Biol. Biochem. 55, 70–77. 10.1016/j.soilbio.2012.06.008

[B24] Fierer N. Strickland M. S. Liptzin D. Bradford M. A. Cleveland C. C. (2009). Global patterns in belowground communities. Ecol. Lett. 12, 1238–1249. 10.1111/j.1461-0248.2009.01360.x 19674041

[B25] Ganesh S. Parris D. J. DeLong E. F. Stewart F. J. (2014). Metagenomic analysis of size-fractionated picoplankton in a marine oxygen minimum zone. ISME J. 8, 187–211. 10.1038/ismej.2013.144 24030599 PMC3869020

[B26] Hamady M. Walker J. J. Harris J. K. Gold N. J. Knight R. (2008). Error-correcting barcoded primers for pyrosequencing hundreds of samples in multiplex. Nat. Methods 5, 235–237. 10.1038/nmeth.1184 18264105 PMC3439997

[B27] Huse S. M. Huber J. A. Morrison H. G. Sogin M. L. Welch D. M. (2007). Accuracy and quality of massively parallel DNA pyrosequencing. Genome Biol. 8:R143. 10.1186/gb-2007-8-7-r143 17659080 PMC2323236

[B28] Jenkinson D. S. Powlson D. S. (1976). Effects of biocidal treatments on metabolism in soil. V. Method for measuring soil biomass. Soil Biol. Biochem. 8, 209–213. 10.1016/0038-0717(76)90005-5

[B29] Jing Z. Cheng J. Jin J. Su J. Bai Y. (2014). Revegetation as an efficient means of improving the diversity and abundance of soil eukaryotes in the Loess Plateau of China. Ecol. Eng. 70, 169–174. 10.1016/j.ecoleng.2014.05.011

[B30] Karst J. Piculell B. Brigham C. Booth M. Hoeksema J. D. (2013). Fungal communities in soils along a vegetative ecotone. Mycologia 105, 61–70. 10.3852/12-042 22802393

[B31] Kemmitt S. J. Lanyon C. V. Waite I. S. Wen Q. Addiscott T. M. Bird N. R. A. (2008). Mineralization of native soil organic matter is not regulated by the size, activity or composition of the soil microbial biomass–a new perspective. Soil Biol. Biochem. 40, 61–73. 10.1016/j.soilbio.2007.06.021

[B32] Kis-Papo T. Grishkan I. Oren A. Wasser S. P. Nevo E. (2001). Spatiotemporal diversity of filamentous fungi in the hypersaline Dead Sea. Mycol. Res. 105, 749–756. 10.1017/S0953756201004129

[B33] Klaubauf S. Inselsbacher E. Zechmeister-Boltenstern S. Wanek W. Gottsberger R. Strauss J.. (2010). Molecular diversity of fungal communities in agricultural soils from Lower Austria. Fungal Divers. 44, 65–75. 10.1007/s13225-010-0053-1 23794962 PMC3688302

[B34] Lara E. Berney C. Harms H. Chatzinotas A. (2007). Cultivation-independent analysis reveals a shift in ciliate 18S rRNA gene diversity in a polycyclic aromatic hydrocarbon-polluted soil. FEMS Microbiol. Ecol. 62, 365–373. 10.1111/j.1574-6941.2007.00387.x 17949434

[B35] Lentendu G. Wubet T. Chatzinotas A. Wilhelm C. Buscot F. Schlegel M. (2014). Effects of long-term differential fertilization on eukaryotic microbial communities in an arable soil: a multiple barcoding approach. Mol. Ecol. 23, 3341–3355. 10.1111/mec.12819 24888892

[B36] Lozupone C. Knight R. (2005). UniFrac: a new phylogenetic method for comparing microbial communities. Appl. Environ. Microb. 71, 8228–8235. 10.1128/AEM.71.12.8228-8235.2005 16332807 PMC1317376

[B37] Lu R. K. (eds.). (2000). Analytical Methods for Soil and Agro-chemistry. Beijing: China Agricultural Science and Technology Press.

[B38] Lundberg D. S. Lebeis S. L. Paredes S. H. Yourstone S. Gehring J. Malfatti S.. (2012). Defining the core *Arabidopsis thaliana* root microbiome. Nature 488, 86–94. 10.1038/nature11237 22859206 PMC4074413

[B39] Lundell T. K. Mäkelä M. R. Hildén K. (2010). Lignin-modifying enzymes in filamentous basidiomycetes–ecological, functional and phylogenetic review. J. Basic Microb. 50, 5–20. 10.1002/jobm.200900338 20175122

[B40] Marschner P. Kandeler E. Marschner B. (2003). Structure and function of the soil microbial community in a long-term fertilizer experiment. Soil Biol. Biochem. 35, 453–461. 10.1016/S0038-0717(02)00297-3

[B41] Moon-van der Staay S. Y. Tzeneva V. A. van der Staay G. W. M. de Vos W. M. Smidt H. Hackstein J. H. P. (2006). Eukaryotic diversity in historical soil samples. FEMS Microbiol. Ecol. 57, 420–428. 10.1111/j.1574-6941.2006.00130.x 16907756

[B42] Morris S. J. Blackwood C. B. (2007). The ecology of soil organisms, in *Soil Microbiology, Ecology, and Biochemistry*, ed Paul E. (Amsterdam: Elsevier), 195–229. 10.1016/B978-0-08-047514-1.50012-3

[B43] Nai C. Wong H. Y. Pannenbecker A. Broughton W. J. Benoit I. de Vries R. P.. (2013). Nutritional physiology of a rock-inhabiting, model microcolonial fungus from an ancestral lineage of the Chaetothyriales (Ascomycetes). Fungal Genet. Biol. 56, 54–66. 10.1016/j.fgb.2013.04.001 23587800

[B44] Nannipieri P. Giagnoni L. Renella G. Puglisi E. Ceccanti B. Masciandaro G. (2012). Soil enzymology: classical and molecular approaches. Biol. Fertil. Soils 48, 743–762. 10.1007/s00374-012-0723-0

[B45] Nelson D. W. Sommers L. E. (1982). Total carbon, organic carbon and organic matter, in Methods of Soil Analysis, Part 2, eds Page A. L. Miller R. H. Keeney D. R. (Madison, WI: American Society of Agronomy), 539–579.

[B46] Nemergut D. R. Townsend A. R. Sattin S. R. Freeman K. R. Fierer N. Neff J. C.. (2008). The effects of chronic nitrogen fertilization on alpine tundra soil microbial communities: implications for carbon and nitrogen cycling. Environ. Microbiol. 10, 3093–3105. 10.1111/j.1462-2920.2008.01735.x 18764871

[B47] Paterson E. (2009). Comments on the regulatory gate hypothesis and implications for C-cycling in soil. Soil Biol. Biochem. 41, 1352–1354. 10.1016/j.soilbio.2009.02.012

[B48] Prober S. M. Leff J. W. Bates S. T. Borer E. T. Firn J. Harpole W. S. (2015). Plant diversity predicts beta but not alpha diversity of soil microbes across grasslands worldwide. Ecol. Lett. 18, 85–95. 10.1111/ele.12381 25430889

[B49] Renella G. Landi L. Nannipieri P. (2002). Hydrolase activities during and after the chloroform fumigation of soil as affected by protease activity. Soil Biol. Biochem. 34, 51–60. 10.1016/S0038-0717(01)00152-3

[B50] Schadt C. W. Martin A. P. Lipson D. A. Schmidt S. K. (2003). Seasonal dynamics of previously unknown fungal lineages in tundra soils. Science 301, 1359–1361. 10.1126/science.1086940 12958355

[B51] Schimel J. P. Schaeffer S. M. (2012). Microbial control over carbon cycling in soil. Front. Microbiol. 3:348. 10.3389/fmicb.2012.00348 23055998 PMC3458434

[B52] Shen C. Liang W. Shi Y. Lin X. Zhang H. Wu X. (2014). Contrasting elevational diversity patterns between eukaryotic soil microbes and plants. Ecology 95, 3190–3202. 10.1890/14-0310.1

[B53] Shi Y. Xiang X. Shen C. Chu H. Neufeld J. D. Walker V. K.. (2015). Vegetation-associated impacts on arctic tundra bacterial and microeukaryotic communities. Appl. Environ. Microb. 81, 492–501. 10.1128/AEM.03229-14 25362064 PMC4277566

[B54] Sterflinger K. Tesei D. Zakharova K. (2012). Fungi in hot and cold deserts with particular reference to microcolonial fungi. Fungal Ecol. 5, 453–462. 10.1016/j.funeco.2011.12.007

[B55] Stres B. Tiedje J. M. (2006). New frontiers in soil microbiology: how to link structure and function of microbial communities? in Nucleic Acids and Proteins in Soil, Vol. 8, eds Nannipieri P. Smalla K. (Berlin: Springer), 1–22. 10.1007/3-540-29449-x_1

[B56] Strope P. K. Nickerson K. W. Harris S. D. Moriyama E. N. (2011). Molecular evolution of urea amidolyase and urea carboxylase in fungi. BMC Evol. Biol. 11:80. 10.1186/1471-2148-11-80 21447149 PMC3073912

[B57] Sugiyama S. Zabed H. M. Okubo A. (2008). Relationships between soil microbial diversity and plant community structure in seminatural grasslands. Grassland Sci. 54, 117–124. 10.1111/j.1744-697X.2008.00113.x

[B58] Tabatabai M. A. (1994). Soil enzymes, in Methods of Soil Analysis, Part 2: Microbiological and Biochemical Properties, eds Weaver R. W. Angle S. Bottomley P. Bezdicek D. Smith S. Tabatabai M. A. (Madison, WI: Soil Science Society of America), 775–833.

[B59] Tu Q. Yuan M. He Z. Deng Y. Xue K. Wu L.. (2015). Fungal communities respond to long-term CO2 elevation by community reassembly. Appl. Environ. Microb. 81, 2445–2454. 10.1128/AEM.04040-14 25616796 PMC4357938

[B60] Tzeneva V. A. Salles J. F. Naumova N. de Vos W. M. Kuikman P. J. Dolfing J.. (2009). Effect of soil sample preservation, compared to the effect of other environmental variables, on bacterial and eukaryotic diversity. Res. Microbiol. 160, 89–98. 10.1016/j.resmic.2008.12.001 19111612

[B61] Vance E. D. Brookes P. C. Jenkinson D. S. (1987). An extraction method for measuring soil microbial biomass C. Soil Biol. Biochem. 19, 703–707. 10.1016/0038-0717(87)90052-6

[B62] Vandenkoornhuyse P. Baldauf S. L. Leyval C. Straczek J. Young J. P. W. (2002). Evolution–Extensive fungal diversity in plant roots. Science 295, 2051–2051. 10.1126/science.295.5562.2051 11896270

[B63] Waldrop M. P. Balser T. C. Firestone M. K. (2000). Linking microbial community composition to function in a tropical soil. Soil Biol. Biochem. 32, 1837–1846. 10.1016/S0038-0717(00)00157-7

[B64] Wu J. Brookes P. C. Jenkinson D. S. (1996). Evidence for the use of a control in the fumigation-incubation method for measuring microbial biomass carbon in soil. Soil Biol. Biochem. 28, 511–518. 10.1016/0038-0717(95)00193-X

[B65] Wu J. Joergensen R. G. Pommerening B. Chaussod R. Brookes P. C. (1990). Measurement of soil microbial biomass C by fumigation extraction–an automated procedure. Soil Biol. Biochem. 22, 1167–1169. 10.1016/0038-0717(90)90046-3

[B66] Zelles L. Palojarvi A. Kandeler E. VonLutzow M. Winter K. Bai Q. Y. (1997). Changes in soil microbial properties and phospholipid fatty acid fractions after chloroform fumigation. Soil Biol. Biochem. 29, 1325–1336. 10.1016/S0038-0717(97)00062-X

