# Supplementary figures and images for: Responses of soil microeukaryotic communities to short-term fumigation-incubation revealed by MiSeq amplicon sequencing
Source: Front Microbiol. 2015 Oct 20;6:1149. doi: 10.3389/fmicb.2015.01149 (PMC4611156; doi:10.3389/fmicb.2015.01149)

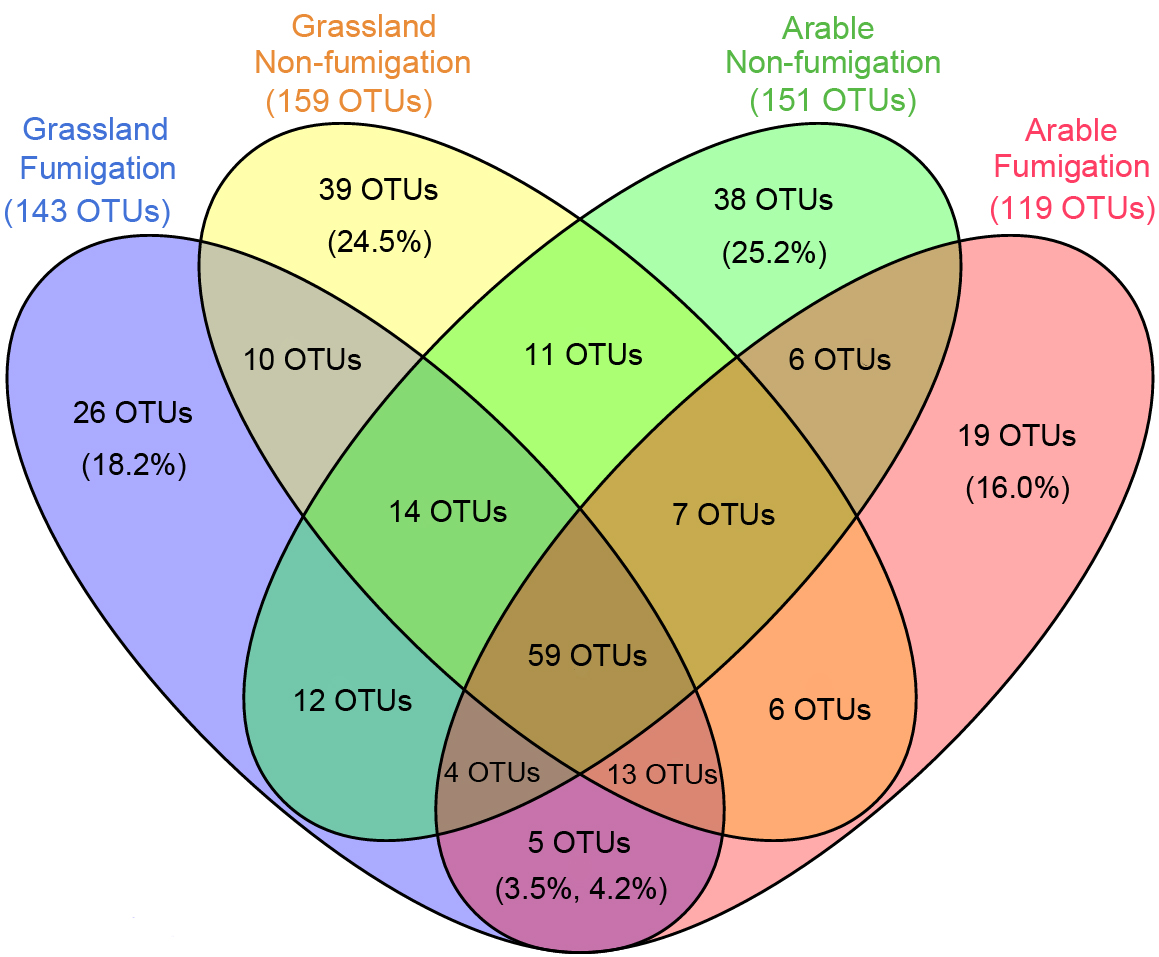

Supplement: Figure S1 — Venn diagram illuminating the amounts of shared and unique OTUs among different treatments at day 30 of the incubation. OTUs were counted from a subset of 5000 sequences per sample, and the average OTUs of three replicates were enumerated. [file Image1.JPEG]

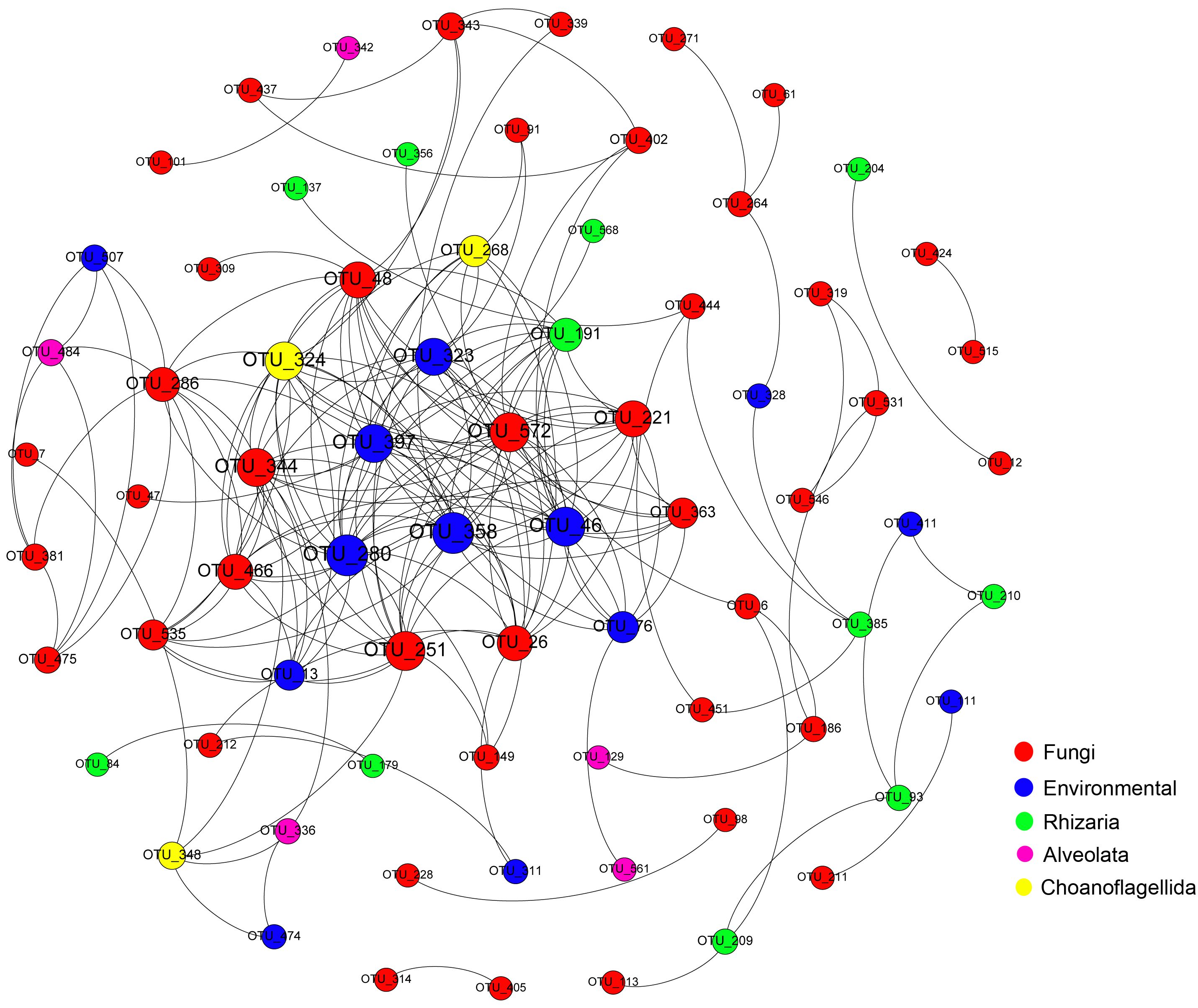

Supplement: Figure S2 — Network analysis on the microeukaryotic communities in the fumigated grassland soil through 30-day incubation. Colored nodes were the OTUs at 97% identity, and the connections indicate significant correlations (r > 0.6, P < 0.01). The size of each node is proportional to the number of connections (degree). [file Image2.JPEG]

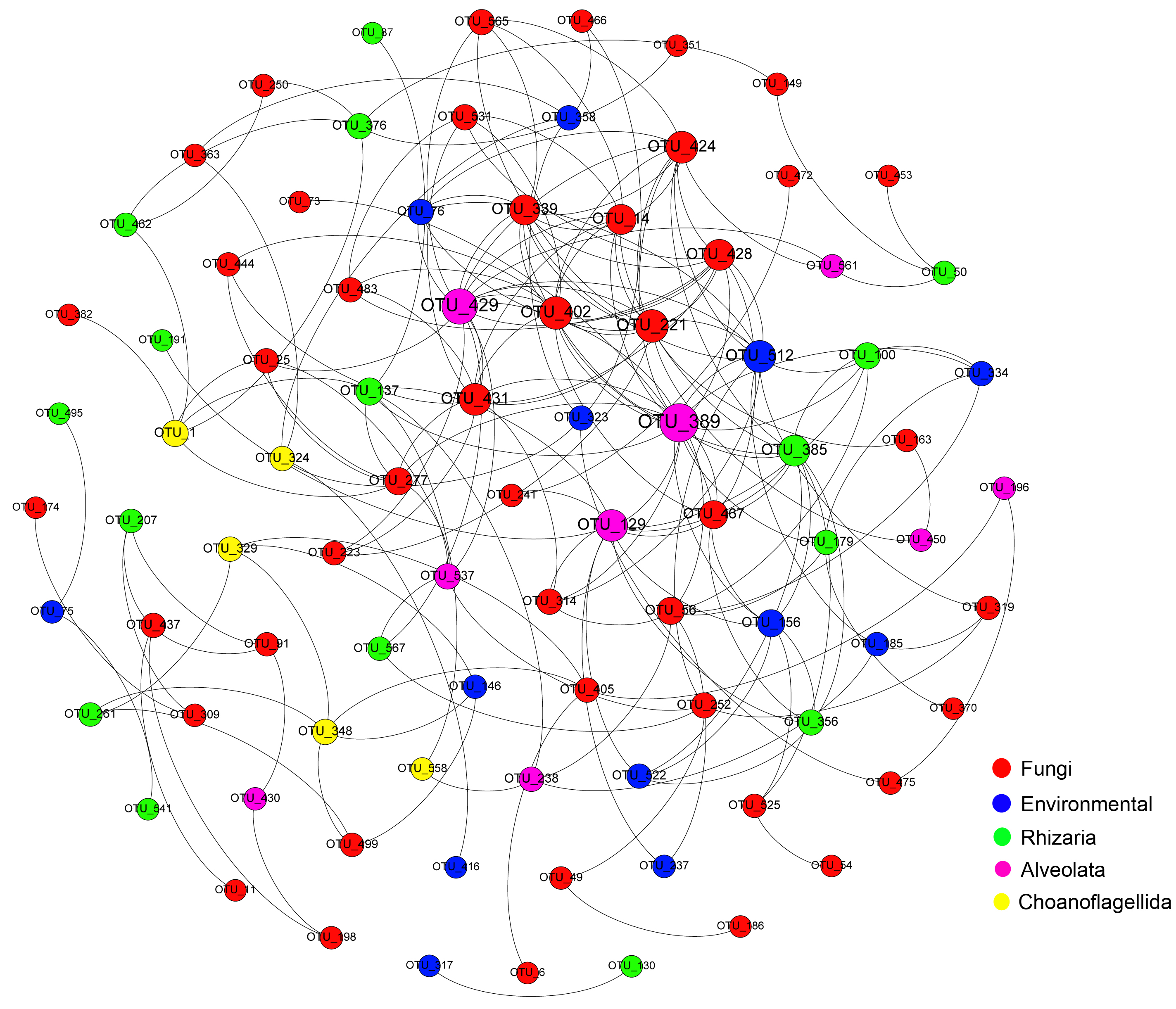

Supplement: Figure S3 — Network analysis on the microeukaryotic communities in the fumigated arable soil through 30-day incubation. Colored nodes were the OTUs at 97% identity, and the connections indicate significant correlations (r > 0.6, P < 0.01). The size of each node is proportional to the number of connections (degree). [file Image3.JPEG]
