# Supplementary material for: Responses of soil microeukaryotic communities to short-term fumigation-incubation revealed by MiSeq amplicon sequencing
Source: Front Microbiol. 2015 Oct 20;6:1149. doi: 10.3389/fmicb.2015.01149 (PMC4611156; doi:10.3389/fmicb.2015.01149)
Supplement: Table S1 — Number of sequences and OTUs measured by QIIME processing. [file Table1.DOCX]

**Supplementary Material**

TABLE S1. Number of sequences and OTUs measured by QIIME processing.

|  | Accession | Count | | |
| --- | --- | --- | --- | --- |
|  |  | Sequence | OTU | Normalized OTU |
| G-F-0 | SAMN03751795 | 13146 | 207 | 141 |
|  | SAMN03751796 | 14395 | 130 | 100 |
|  | SAMN03751797 | 11411 | 120 | 99 |
| A-F-0 | SAMN03751798 | 14349 | 203 | 138 |
|  | SAMN03751799 | 11679 | 206 | 149 |
|  | SAMN03751800 | 21203 | 229 | 140 |
| G-0 | SAMN03751801 | 3485 | 95 | missed |
|  | SAMN03751802 | 5778 | 134 | 126 |
|  | SAMN03751803 | 5162 | 153 | 151 |
| A-0 | SAMN03751804 | 22228 | 270 | 151 |
|  | SAMN03751805 | 17805 | 240 | 145 |
|  | SAMN03751806 | 12423 | 216 | 149 |
| G-F-7 | SAMN03751807 | 13912 | 142 | 104 |
|  | SAMN03751808 | 15729 | 141 | 103 |
|  | SAMN03751809 | 10000 | 122 | 102 |
| A-F-7 | SAMN03751810 | 21083 | 148 | 80 |
|  | SAMN03751811 | 14543 | 196 | 128 |
|  | SAMN03751812 | 15797 | 219 | 142 |
| G-7 | SAMN03751813 | 5782 | 131 | 123 |
|  | SAMN03751814 | 6649 | 155 | 142 |
|  | SAMN03751815 | 5360 | 127 | 123 |
| A-7 | SAMN03751816 | 12422 | 205 | 151 |
|  | SAMN03751817 | 22373 | 259 | 156 |
|  | SAMN03751818 | 19546 | 246 | 150 |
| G-F-30 | SAMN03751819 | 15042 | 172 | 132 |
|  | SAMN03751820 | 12662 | 203 | 150 |
|  | SAMN03751821 | 8737 | 177 | 144 |
| A-F-30 | SAMN03751822 | 18287 | 176 | 103 |
|  | SAMN03751823 | 16767 | 250 | 159 |
|  | SAMN03751824 | 11724 | 133 | 94 |
| G-30 | SAMN03751825 | 14487 | 234 | 159 |
|  | SAMN03751826 | 14592 | 244 | 174 |
|  | SAMN03751827 | 7467 | 162 | 142 |
| A-30 | SAMN03751828 | 24947 | 259 | 138 |
|  | SAMN03751829 | 8655 | 183 | 141 |
|  | SAMN03751830 | 5355 | 177 | 173 |

G, grassland soil; A, arable soil; F, fumigation; number, incubation days. OTU counts were normalized based on the randomly selected 5,000 sequences per sample.

TABLE S2. Relative frequency (%) of the main subphyla affiliated with Ascomycota and Basidiomycota.

|  | Accession | Ascomycota | | |  | Basidiomycota | | | |
| --- | --- | --- | --- | --- | --- | --- | --- | --- | --- |
|  |  | Pezizom-  ycotina | Saccharo-  mycotina | Mitosporic  Ascomycota |  | Agarico-  mycotina | Incertae  sedis | Puccinio-  mycotina | Ustilagin-  omycotina |
| G-F-0 | SAMN03751795 | 54.1 | 0.1 | 0.3 |  | 3.2 | 0.2 | 0.5 | 0.0 |
|  | SAMN03751796 | 45.8 | 0.2 | 0.2 |  | 1.8 | 0.1 | 0.7 | 0.2 |
|  | SAMN03751797 | 58.0 | 0.1 | 0.5 |  | 1.8 | 0.4 | 0.6 | 0.2 |
| A-F-0 | SAMN03751798 | 57.6 | 0.6 | 0.7 |  | 1.6 | 0.0 | 0.1 | 0.1 |
|  | SAMN03751799 | 52.8 | 0.8 | 1.0 |  | 3.0 | 0.0 | 0.2 | 0.1 |
|  | SAMN03751800 | 54.4 | 1.3 | 1.3 |  | 2.4 | 0.0 | 0.6 | 0.2 |
| G-0 | SAMN03751801 | 61.3 | 0.1 | 0.2 |  | 2.7 | 0.1 | 0.3 | 0.1 |
|  | SAMN03751802 | 73.6 | 0.7 | 0.7 |  | 2.2 | 0.3 | 0.1 | 0.1 |
|  | SAMN03751803 | 51.0 | 0.1 | 0.4 |  | 3.5 | 0.2 | 0.5 | 0.0 |
| A-0 | SAMN03751804 | 52.2 | 0.6 | 0.8 |  | 2.6 | 0.0 | 0.1 | 0.1 |
|  | SAMN03751805 | 61.2 | 0.8 | 0.5 |  | 1.6 | 0.0 | 0.1 | 0.1 |
|  | SAMN03751806 | 51.4 | 0.6 | 0.7 |  | 1.7 | 0.0 | 0.0 | 0.1 |
| G-F-7 | SAMN03751807 | 53.1 | 0.1 | 0.4 |  | 1.0 | 0.0 | 0.6 | 0.3 |
|  | SAMN03751808 | 45.3 | 0.0 | 0.5 |  | 1.3 | 0.2 | 0.9 | 0.0 |
|  | SAMN03751809 | 45.0 | 0.2 | 0.2 |  | 4.1 | 0.1 | 1.0 | 0.1 |
| A-F-7 | SAMN03751810 | 91.3 | 0.1 | 0.1 |  | 0.4 | 0.0 | 0.0 | 0.1 |
|  | SAMN03751811 | 69.1 | 0.4 | 0.7 |  | 1.5 | 0.0 | 0.1 | 0.1 |
|  | SAMN03751812 | 51.9 | 0.5 | 0.9 |  | 2.2 | 0.0 | 0.2 | 0.1 |
| G-7 | SAMN03751813 | 53.5 | 0.1 | 0.1 |  | 0.6 | 0.1 | 0.1 | 0.0 |
|  | SAMN03751814 | 42.2 | 0.0 | 0.0 |  | 1.8 | 0.3 | 0.0 | 0.3 |
|  | SAMN03751815 | 38.2 | 0.1 | 0.1 |  | 1.9 | 0.0 | 0.3 | 0.2 |
| A-7 | SAMN03751816 | 62.9 | 0.6 | 0.7 |  | 1.7 | 0.0 | 0.1 | 0.1 |
|  | SAMN03751817 | 56.2 | 0.5 | 0.7 |  | 2.0 | 0.0 | 0.2 | 0.1 |
|  | SAMN03751818 | 58.2 | 0.7 | 0.9 |  | 2.1 | 0.0 | 0.0 | 0.1 |
| G-F-30 | SAMN03751819 | 56.0 | 0.1 | 0.4 |  | 1.1 | 0.0 | 0.2 | 0.0 |
|  | SAMN03751820 | 60.2 | 0.2 | 0.5 |  | 1.5 | 0.2 | 0.1 | 0.0 |
|  | SAMN03751821 | 56.1 | 0.0 | 0.5 |  | 1.4 | 0.1 | 0.5 | 0.1 |
| A-F-30 | SAMN03751822 | 88.0 | 0.0 | 0.2 |  | 0.5 | 0.0 | 0.0 | 0.0 |
|  | SAMN03751823 | 70.4 | 0.2 | 0.3 |  | 2.1 | 0.1 | 0.1 | 0.0 |
|  | SAMN03751824 | 87.7 | 0.0 | 0.2 |  | 0.4 | 0.0 | 0.0 | 0.0 |
| G-30 | SAMN03751825 | 57.1 | 0.2 | 0.4 |  | 1.7 | 0.1 | 0.2 | 0.0 |
|  | SAMN03751826 | 58.3 | 0.6 | 0.7 |  | 2.1 | 0.1 | 0.1 | 0.0 |
|  | SAMN03751827 | 49.3 | 0.1 | 0.3 |  | 1.4 | 0.1 | 0.1 | 0.1 |
| A-30 | SAMN03751828 | 65.2 | 0.4 | 0.9 |  | 1.1 | 0.0 | 0.0 | 0.0 |
|  | SAMN03751829 | 68.8 | 0.7 | 0.8 |  | 1.5 | 0.0 | 0.0 | 0.0 |
|  | SAMN03751830 | 57.2 | 0.4 | 0.8 |  | 1.1 | 0.1 | 0.1 | 0.1 |

G, grassland soil; A, arable soil; F, fumigation; number, incubation days.

TABLE S3. Relative frequency (%) of the main identifiable taxa.

|  | Accession | Eimer-  iorina | Chytri-  diaceae | Dothide-  omycetes | Eurotio-  mycetes | Laboulbeniomycetes | Sordario-mycetes | Saccharo-mycetes | Conio-  sporium | Scolecob-asidium | Agarico-mycetes | Tremello-mycetes | Microbotr-yomycetes | Tritira-  chium | Ustilagin-  omycetes | Kickx-  ellales | Morti-  erellales | Muc-  orales | Zoop-  agales | Glom-  eraceae | Uncultured Chytridiomycota |
| --- | --- | --- | --- | --- | --- | --- | --- | --- | --- | --- | --- | --- | --- | --- | --- | --- | --- | --- | --- | --- | --- |
| G-F-0 | SAMN03751795 | 5.5 | 0.1 | 0.4 | 29.0 | 0.1 | 24.7 | 0.1 | 0.1 | 0.0 | 0.7 | 2.4 | 0.3 | 0.2 | 0.0 | 0.7 | 1.5 | 1.9 | 0.1 | 0.2 | 1.1 |
|  | SAMN03751796 | 6.5 | 0.1 | 0.3 | 27.9 | 0.0 | 17.6 | 0.2 | 0.0 | 0.0 | 0.1 | 1.6 | 0.5 | 0.2 | 0.2 | 0.7 | 1.6 | 0.2 | 0.1 | 0.0 | 3.5 |
|  | SAMN03751797 | 3.8 | 0.1 | 0.4 | 36.4 | 0.0 | 21.3 | 0.1 | 0.0 | 0.0 | 0.1 | 1.7 | 0.5 | 0.1 | 0.2 | 1.0 | 1.4 | 0.7 | 0.1 | 0.0 | 0.3 |
| A-F-0 | SAMN03751798 | 19.3 | 0.2 | 0.3 | 9.7 | 0.1 | 47.5 | 0.6 | 0.4 | 0.1 | 0.1 | 1.5 | 0.1 | 0.0 | 0.1 | 0.0 | 0.4 | 1.1 | 0.2 | 0.5 | 0.6 |
|  | SAMN03751799 | 18.0 | 0.2 | 0.3 | 7.0 | 0.1 | 45.3 | 0.8 | 0.6 | 0.2 | 0.1 | 2.9 | 0.2 | 0.0 | 0.1 | 0.2 | 0.8 | 0.7 | 0.4 | 0.2 | 0.8 |
|  | SAMN03751800 | 18.2 | 0.3 | 0.4 | 4.6 | 0.1 | 49.3 | 1.3 | 1.0 | 0.1 | 0.2 | 2.2 | 0.5 | 0.0 | 0.2 | 0.1 | 0.8 | 0.6 | 0.2 | 0.3 | 0.9 |
| G-0 | SAMN03751801 | 10.7 | 0.1 | 0.1 | 25.0 | 0.1 | 36.0 | 0.1 | 0.0 | 0.1 | 0.1 | 2.6 | 0.3 | 0.0 | 0.1 | 0.0 | 0.4 | 0.9 | 0.0 | 1.0 | 1.1 |
|  | SAMN03751802 | 4.5 | 0.1 | 0.1 | 28.0 | 0.1 | 45.4 | 0.7 | 0.5 | 0.1 | 0.3 | 1.9 | 0.1 | 0.0 | 0.1 | 0.5 | 0.6 | 1.3 | 0.1 | 1.4 | 0.4 |
|  | SAMN03751803 | 15.2 | 1.0 | 0.2 | 15.8 | 0.0 | 35.0 | 0.1 | 0.1 | 0.0 | 0.5 | 3.0 | 0.5 | 0.0 | 0.0 | 0.3 | 0.8 | 1.7 | 0.0 | 4.0 | 1.0 |
| A-0 | SAMN03751804 | 23.0 | 0.2 | 0.3 | 5.9 | 0.2 | 45.8 | 0.6 | 0.4 | 0.1 | 0.2 | 2.4 | 0.1 | 0.0 | 0.1 | 0.1 | 0.5 | 1.3 | 0.2 | 0.2 | 0.7 |
|  | SAMN03751805 | 18.0 | 0.3 | 0.3 | 5.8 | 0.1 | 54.9 | 0.8 | 0.2 | 0.1 | 0.1 | 1.6 | 0.1 | 0.0 | 0.1 | 0.1 | 0.5 | 0.6 | 0.2 | 0.3 | 0.9 |
|  | SAMN03751806 | 24.4 | 0.2 | 0.3 | 4.3 | 0.1 | 46.7 | 0.6 | 0.5 | 0.1 | 0.1 | 1.6 | 0.0 | 0.0 | 0.1 | 0.2 | 0.7 | 0.7 | 0.1 | 0.4 | 1.4 |
| G-F-7 | SAMN03751807 | 3.5 | 0.1 | 0.5 | 28.5 | 0.1 | 24.0 | 0.1 | 0.0 | 0.0 | 0.1 | 0.8 | 0.4 | 0.1 | 0.3 | 0.5 | 0.4 | 0.4 | 0.0 | 0.1 | 0.3 |
|  | SAMN03751808 | 5.0 | 0.1 | 0.5 | 24.0 | 0.0 | 20.8 | 0.0 | 0.0 | 0.0 | 0.1 | 1.3 | 0.5 | 0.5 | 0.0 | 1.1 | 0.5 | 0.6 | 0.1 | 0.1 | 0.5 |
|  | SAMN03751809 | 5.5 | 0.1 | 0.4 | 26.0 | 0.0 | 18.6 | 0.2 | 0.0 | 0.0 | 0.1 | 4.0 | 0.5 | 0.5 | 0.1 | 1.6 | 0.6 | 0.6 | 0.1 | 0.4 | 0.4 |
| A-F-7 | SAMN03751810 | 3.3 | 0.1 | 0.1 | 83.7 | 0.0 | 7.5 | 0.1 | 0.1 | 0.0 | 0.0 | 0.4 | 0.0 | 0.0 | 0.1 | 0.1 | 0.1 | 0.1 | 0.0 | 0.1 | 0.2 |
|  | SAMN03751811 | 9.0 | 0.2 | 0.2 | 33.9 | 0.0 | 34.9 | 0.4 | 0.5 | 0.1 | 0.1 | 1.4 | 0.1 | 0.0 | 0.1 | 0.1 | 0.5 | 0.4 | 0.0 | 0.2 | 0.7 |
|  | SAMN03751812 | 15.2 | 0.2 | 0.3 | 17.6 | 0.1 | 33.8 | 0.5 | 0.5 | 0.2 | 0.2 | 2.0 | 0.1 | 0.0 | 0.1 | 0.3 | 0.6 | 0.5 | 0.1 | 0.2 | 0.9 |
| G-7 | SAMN03751813 | 4.2 | 0.0 | 0.1 | 13.8 | 0.0 | 39.6 | 0.1 | 0.1 | 0.0 | 0.1 | 0.6 | 0.1 | 0.0 | 0.0 | 0.2 | 0.8 | 0.4 | 0.0 | 1.1 | 0.7 |
|  | SAMN03751814 | 5.5 | 0.2 | 0.0 | 11.4 | 0.0 | 30.9 | 0.0 | 0.0 | 0.0 | 0.5 | 1.4 | 0.0 | 0.0 | 0.3 | 0.0 | 0.2 | 0.0 | 0.0 | 0.3 | 13.7 |
|  | SAMN03751815 | 8.9 | 0.4 | 0.2 | 11.7 | 0.0 | 26.3 | 0.1 | 0.0 | 0.0 | 0.1 | 1.8 | 0.3 | 0.0 | 0.2 | 0.2 | 0.5 | 2.3 | 0.0 | 1.1 | 1.3 |
| A-7 | SAMN03751816 | 14.1 | 0.3 | 0.3 | 6.4 | 0.1 | 56.1 | 0.6 | 0.4 | 0.1 | 0.2 | 1.5 | 0.1 | 0.0 | 0.1 | 0.0 | 0.6 | 0.7 | 0.1 | 0.1 | 0.6 |
|  | SAMN03751817 | 17.1 | 0.3 | 0.3 | 4.6 | 0.0 | 51.1 | 0.5 | 0.4 | 0.1 | 0.3 | 1.7 | 0.1 | 0.0 | 0.1 | 0.0 | 0.5 | 0.8 | 0.1 | 0.2 | 0.5 |
|  | SAMN03751818 | 16.5 | 0.4 | 0.3 | 4.5 | 0.1 | 53.3 | 0.7 | 0.5 | 0.1 | 0.1 | 2.0 | 0.0 | 0.0 | 0.1 | 0.3 | 0.5 | 0.7 | 0.1 | 0.7 | 0.4 |
| G-F-30 | SAMN03751819 | 5.5 | 0.1 | 0.2 | 15.5 | 0.1 | 40.2 | 0.1 | 0.3 | 0.1 | 0.1 | 1.0 | 0.1 | 0.0 | 0.0 | 0.1 | 1.1 | 1.5 | 0.0 | 4.2 | 0.7 |
|  | SAMN03751820 | 6.1 | 0.1 | 0.2 | 20.4 | 0.2 | 39.4 | 0.2 | 0.2 | 0.1 | 0.2 | 1.4 | 0.0 | 0.0 | 0.0 | 0.2 | 0.8 | 1.4 | 0.0 | 2.8 | 0.8 |
|  | SAMN03751821 | 5.9 | 0.2 | 0.3 | 18.2 | 0.1 | 37.5 | 0.0 | 0.3 | 0.1 | 0.2 | 1.2 | 0.3 | 0.1 | 0.1 | 0.0 | 0.8 | 1.0 | 0.0 | 2.9 | 0.7 |
| A-F-30 | SAMN03751822 | 2.3 | 0.1 | 0.1 | 74.5 | 0.0 | 13.4 | 0.0 | 0.1 | 0.0 | 0.0 | 0.4 | 0.0 | 0.0 | 0.0 | 0.0 | 0.3 | 0.2 | 0.0 | 0.8 | 0.2 |
|  | SAMN03751823 | 6.6 | 0.4 | 0.2 | 40.2 | 0.1 | 29.9 | 0.2 | 0.2 | 0.1 | 0.5 | 1.6 | 0.1 | 0.0 | 0.0 | 0.0 | 0.2 | 1.1 | 0.1 | 2.2 | 0.4 |
|  | SAMN03751824 | 2.0 | 0.0 | 0.0 | 72.5 | 0.0 | 15.1 | 0.0 | 0.1 | 0.0 | 0.0 | 0.4 | 0.0 | 0.0 | 0.0 | 0.0 | 0.3 | 0.3 | 0.0 | 1.2 | 0.3 |
| G-30 | SAMN03751825 | 6.0 | 0.3 | 0.2 | 19.1 | 0.1 | 37.7 | 0.2 | 0.3 | 0.1 | 0.3 | 1.4 | 0.2 | 0.0 | 0.0 | 0.1 | 0.7 | 0.9 | 0.0 | 1.9 | 0.8 |
|  | SAMN03751826 | 6.8 | 0.3 | 0.2 | 17.8 | 0.1 | 40.1 | 0.6 | 0.5 | 0.1 | 0.5 | 1.6 | 0.1 | 0.0 | 0.0 | 0.0 | 0.6 | 1.0 | 0.1 | 1.9 | 0.7 |
|  | SAMN03751827 | 5.0 | 0.1 | 0.1 | 18.7 | 0.1 | 30.3 | 0.1 | 0.2 | 0.1 | 0.1 | 1.4 | 0.1 | 0.0 | 0.0 | 0.0 | 0.5 | 1.0 | 0.1 | 1.8 | 2.2 |
| A-30 | SAMN03751828 | 8.9 | 0.0 | 0.2 | 6.1 | 0.0 | 58.8 | 0.4 | 0.5 | 0.3 | 0.0 | 1.1 | 0.0 | 0.0 | 0.0 | 0.0 | 0.5 | 0.6 | 0.1 | 0.3 | 0.6 |
|  | SAMN03751829 | 10.2 | 0.1 | 0.2 | 6.4 | 0.1 | 62.1 | 0.7 | 0.6 | 0.1 | 0.1 | 1.4 | 0.0 | 0.0 | 0.0 | 0.1 | 0.3 | 0.5 | 0.1 | 0.5 | 1.1 |
|  | SAMN03751830 | 9.5 | 0.2 | 0.2 | 13.7 | 0.1 | 43.2 | 0.4 | 0.5 | 0.2 | 0.2 | 1.0 | 0.0 | 0.0 | 0.1 | 0.0 | 0.6 | 1.0 | 0.0 | 2.6 | 1.1 |

The microeukaryotic taxa in which the relative frequencies exceeded 0.1% in at least one treatment were selected. G, grassland soil; A, arable soil; F, fumigation; number, incubation days.
